# A Review of Natural Polysaccharides: Sources, Characteristics, Properties, Food, and Pharmaceutical Applications

**DOI:** 10.3390/ijms25021322

**Published:** 2024-01-22

**Authors:** Ikbel Benalaya, Gilberto Alves, João Lopes, Luís R. Silva

**Affiliations:** 1 CICS-UBI—Health Sciences Research Centre, University of Beira Interior, 6201-001 Covilha, Portugal; ikbela94@gmail.com (I.B.); gilberto@fcsaude.ubi.pt (G.A.); 2 iMed.ULisboa, Research Institute for Medicines, Faculdade de Farmácia, University of Lisboa, 1649-003 Lisbon, Portugal; 3 CPIRN-UDI/IPG, Center of Potential and Innovation of Natural Resources, Research Unit for Inland Development (UDI), Polytechnic Institute of Guarda, 6300-559 Guarda, Portugal; 4 CIEPQPF, Department of Chemical Engineering, Pólo II—Pinhal de Marrocos, University of Coimbra, 3030-790 Coimbra, Portugal

**Keywords:** polymers biopolymers, excipient, natural source, food and pharmaceutical applications

## Abstract

Natural polysaccharides, which are described in this study, are some of the most extensively used biopolymers in food, pharmaceutical, and medical applications, because they are renewable and have a high level of biocompatibility and biodegradability. The fundamental understanding required to properly exploit polysaccharides potential in the biocomposite, nanoconjugate, and pharmaceutical industries depends on detailed research of these molecules. Polysaccharides are preferred over other polymers because of their biocompatibility, bioactivity, homogeneity, and bioadhesive properties. Natural polysaccharides have also been discovered to have excellent rheological and biomucoadhesive properties, which may be used to design and create a variety of useful and cost-effective drug delivery systems. Polysaccharide-based composites derived from natural sources have been widely exploited due to their multifunctional properties, particularly in drug delivery systems and biomedical applications. These materials have achieved global attention and are in great demand because to their biochemical properties, which mimic both human and animal cells. Although synthetic polymers account for a substantial amount of organic chemistry, natural polymers play a vital role in a range of industries, including biomedical, pharmaceutical, and construction. As a consequence, the current study will provide information on natural polymers, their biological uses, and food and pharmaceutical applications.

## 1. Introduction

**Figure 1:**
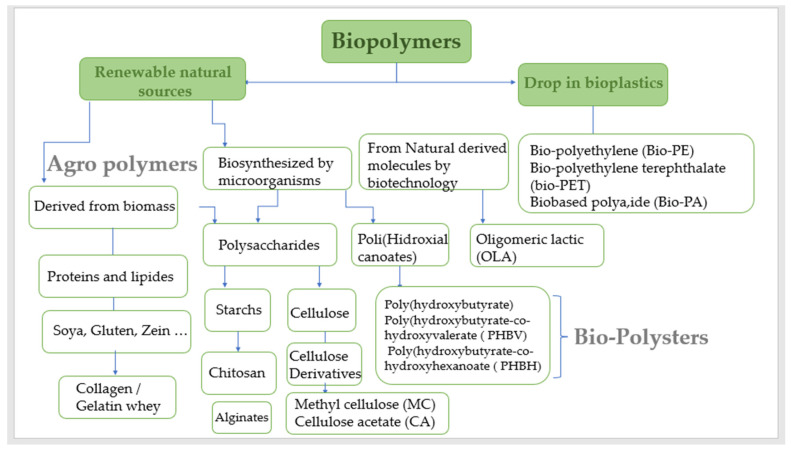
Classification of natural polymers.

Over the last several decades, more sustainable plastics have been developed with the objective of reducing the use of non-renewable resources in their production. This is due to increased ecological knowledge about the need to reduce the environmental impact that conventional plastics have on the environment (Figure 1). The goal of sustainable development is to replace conventional petroleum-based polymers with more environmentally friendly materials in a variety of industrial sectors. As a result, increased production and use of bio-based polymers in recent years has placed biopolymers as one of the most promising ways to achieve this goal. Valorization of agro-food wastes, biopolymers produced by yeast, algae, or bacterial fermentation, as well as those extracted directly from biomass, such as polysaccharides, proteins, and lipids, have all been discussed and created a lot of interest, especially for medical products, food packaging, agricultural films, membrane process applications, sustainable clothing, and other areas that, despite recent successful developments in bio-based polymers, continue to require improvement [1].

Depending on their natural origin, natural polymers can be divided into six major groups: polysaccharides, proteins, polynucleotides, polyesters, lignin, and polyisoprenes [2].

Polysaccharides and polypeptides are frequently employed as drug carriers in cancer therapy. Excellent biocompatibility, accessibility, stability, non-toxicity, cost-effectiveness, and biodegradability are a part of natural polymers, particularly those based on polysaccharides [3]. Several polysaccharides, including starch, dextran, pullulan, alginate, pectin, chitin, chitosan, hyaluronic acid, albumin, gelatin, and guar gum, are employed to produce nanocarriers for cancer therapy, according to the literature. 

Polyelectrolyte complexation, hydrophobically modified polymer self-assembly, covalent or ionic cross-linking, and other processes are used to create polysaccharide-based nanoparticles [4]. Several natural polymers have been found to exhibit anticancer properties on their own. Chitosan, for examples, has anticancer properties because it may perforate the membranes of tumor cells and induce apoptosis. Several papers have extensively investigated the anticancer potential of chitosan against sarcomas and hepatocarcinomas [5]. Less soluble treatments have been attached to and transported to tumor locations using natural polymers as a basis.

The high degree of flexibility of these polymers offers the possibility of reducing drug loss and toxicity to healthy tissue.

Most naturally occurring polymers are condensation polymers, which form rapidly as a by-product of the joining of monomer units to produce small molecules (often water). Other polymers are those that are formed without by-products by mixing the monomer units that comprise the polymer. Polymers found in nature may be classified into six broad groups based on their origins: polyesters, lignin, polyisoprenes, polysaccharides, polynucleotides, and proteins [6]. Polysaccharides and proteins have both been extensively investigated in the DDS due to their biocompatibility and processability. Natural biopolymers can be produced by algae, plants, animals, bacteria, and microorganisms. This article discusses several polymers produced by plants, animals, and microorganisms [7].

Excipients produced from natural polymers are used in pharmaceuticals. Anti-foaming agents, such as silicone, vegetable gums, cellulose, starch, and polyvinyl acetate (PVAc) are used in emulsions. Their major purpose is to prevent unfavorable organoleptic properties [8]. There are three categories of biodegradable biopolymers derived from renewable resources:

1. Plant-based polymers, such as polysaccharides (such as cellulose, starch, chitin, and chitosan) and proteins. 2. Microorganism-derived, such as polyhydroxybutyrate (PHB). 3. Polymers are created indirectly through the polymerization of monomers. These polymers are frequently derived from plant resources, such as lactic acid, and are formed when sugars ferment or when reactive monomeric compounds are derived from vegetable oils combined with sugars. A variety of biopolymers are employed in medical sectors. Polymers used in sutures and medical implants include polylactides (PLA), polyglycolides (PGA), and polylactide-coglycolide co-polymers (PLGA) [9].

These biopolymers are non-toxic and well tolerated by the body. Other biopolymers suitable for pharmaceutical uses include cellulose, polyamino acids, and polyhydroxyal kanoates (PHA) [10].

## 2. Classification of Natural Polysaccharides

Polysaccharides, which belong to the third main class of biopolymers (carbohydrates), are important for the immune system, blood coagulation, fertilization, pathogenesis prevention, and therapeutic effectiveness [11]. Structure support, energy storage, lubrication, and cell signal transduction are only a few of the biological functions that polysaccharides have an impact on in cells [12].

**Figure 2:**
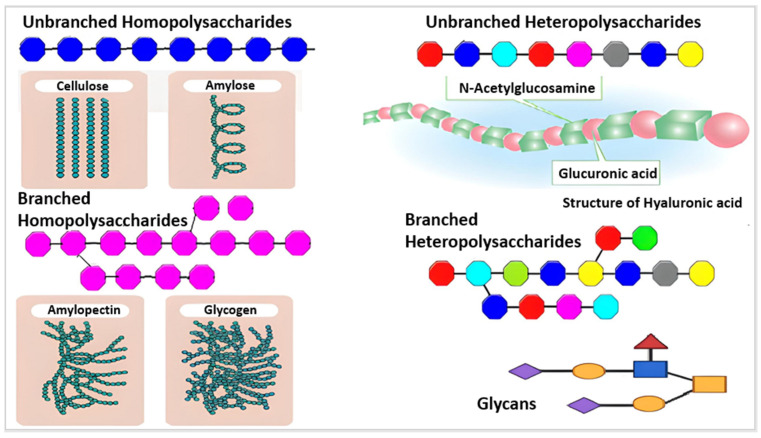
Different monosaccharides are represented by various colors, including branched and unbranched homopolysaccharides and heteropolysaccharides [14].

Based on their chemical structure, which consists of monosaccharide units joined by glycosidic linkages, polysaccharides—the most prevalent type of carbohydrates in nature—are categorized [13]. They may also establish covalent bonds with other structures like lipids, peptides, and amino acids. In contrast to heteropolysaccharides, which are heteroglycans made up of several monosaccharides, homopolysaccharides are homoglycans composed of the same monosaccharide (Figure 2) [12].

**Figure 3:**
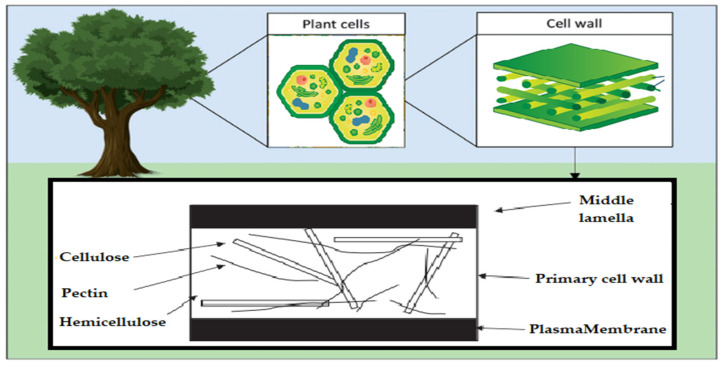
Schematic of the polysaccharide components of the plant cell wall.

Acetic linkages are used to join homopolymers of glucose or amino sugars. Polysaccharides are the world’s most abundant renewable resource, according to current statistics. Each year, photosynthesis produces several orders of magnitude more carbohydrate than is generated artificially (Figure 3) [15].

Polysaccharides are classified into various classes based on their structure or function (Table 1). There are three basic categories: polysaccharides include structural polysaccharides like cellulose and chitin, storage polysaccharides like starch and glycogen, and gel-forming polysaccharides like alginic acid and mucopolysaccharides. Ionic or nonionic (cationic and anionic) polymers can be found as well as branched or straight-chained polymers [16].

**Figure 4:**
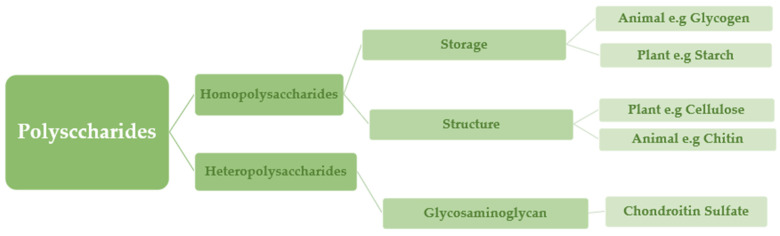
Classification of polysaccharides based on physiological properties and the type of monosaccharides utilized as building blocks.

Natural polysaccharides, such as cellulose (Figure 4), have stability and physical structure, which adds to their ability to retain food, such as starch [14]. Various examples of negatively charged polysaccharides are pectin, heparin, hyaluronic acid, and alginate. Chitosan is a polysaccharide that is positively charged, whereas pectin, pectinol, and alginate are polyelectrolyte-based polysaccharides [12].

### 2.1. Cellulose

Since it constitutes the majority of plant cell walls and almost half of the biomass in photosynthetic species, cellulose may be the most common chemical on the planet. Several materials, including cotton, linen, wood, hemp, jute, kenaf, sugar beet cereal straws, and flax, have long been solid sources of cellulose. Other cellulose sources include bacteria (such as *Acetobacter*), algae (like *Valonia* and *Microdicyon*), and marine animals of the *Ascite* family [17].

**Figure 5:**
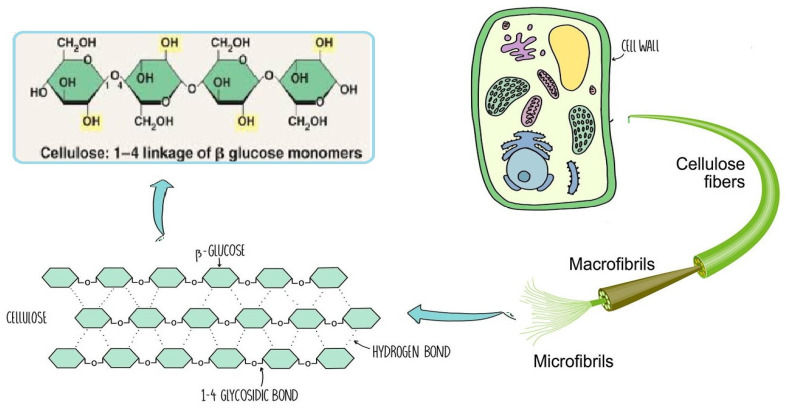
Schematic of cellulose chain.

**Figure 6:**
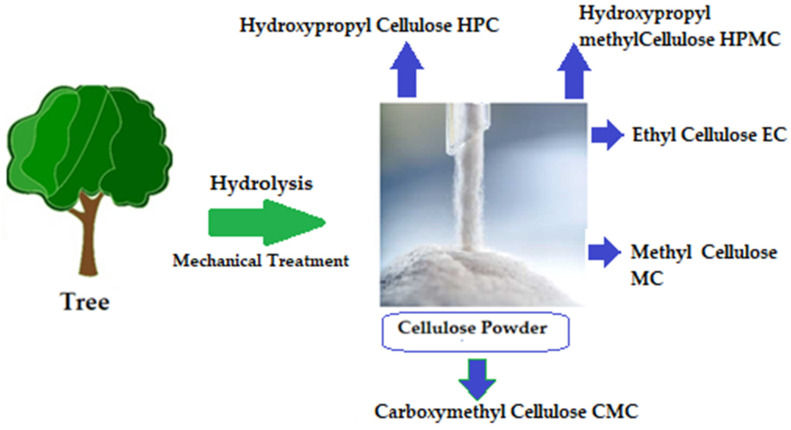
Variety of cellulose ethers derived from different preparation methods [24].

The chemical structure of cellulose is illustrated in Figure 5 as having three hydroxyl groups per AGU, with the exception of the terminal ends. These hydroxyl groups collaborate to generate intra- and inter-hydroxyl hydrogen bonds, resulting in a supramolecular polymer with crystalline (ordered) sections [18]. When cellulose molecules are randomly organized, they form amorphous patches that connect the ordered crystalline areas together [19]. The structure of cellulose has a significant impact on its reactivity. The hydroxyl groups on the surface of the cellulose molecule can create both intramolecular and intermolecular hydrogen bonds [20]. Many of the physical and chemical features of the polymeric chains are dictated by the hydrogen bonds formed between them, which may increase their linear integrity. Nonetheless, cellulose has several intrinsic disadvantages, including low crease resistance, poor solvent solubility, and a lack of thermoplasticity. To improve the properties of cellulose, controlled chemical or physical surface modification is necessary [21]. The biosynthesized component of natural cellulose, in general, cannot be processed as a synthetic polymer [22]. One proposed solution is to surface-functionalize cellulose molecules with foreign groups. This enables the cellulose surface chemistry to be modified without affecting the drug’s various critical intrinsic properties, including self-assembly, controlled dispersion inside a variety of matrix polymers, and increasing particle–particle and particle–matrix bond strengths. Numerous drug delivery methods use conventional cellulose and its derivatives, including cellulose ethers, cellulose esters, and oxycellulose. Sodium carboxymethyl cellulose (CMC), methylcellulose (MC), ethylcellulose (EC), hydroxypropyl cellulose (HPC), and hydroxypropyl methyl cellulose (HPMC) are among the several cellulose ethers that can be found in nature (Figure 6) [23].

### 2.2. Hemicelluloses

Another important component of plant cells is hemicelluloses, which form a matrix around cellulose microfibrils, are composed of a variety of molecules, including xyloglucans, xylans, mannans, and (1–3)-(1–4)-glucans [25,26,27]. Chemical interactions like hydrogen bonds and van der Waals forces are frequently used to link them to the cellulose microfibrils. Hemicelluloses also function as metabolic reserves and signaling molecules in cells [28], with a global yearly production of over 60 billion tons, which is the second-most abundant renewable component of lignocellulosic biomass after cellulose [29,30].

### 2.3. Pectin

Plant cell walls also contain cellulose, hemicelluloses, and lignin in addition to pectin. It belongs to the same group of polysaccharides as agar and mucopolysaccharides which form gels [31]. One of the most crucial hydrocolloids for industry is pectin, which is employed in many different food products. Citrus and apple pectin are the main industrial sources of high-methoxyl (>50%) pectin, whereas sunflower pectin is a naturally occurring source of low-methoxyl (50%) pectin [32].

### 2.4. Starch

Pure starch is an odorless white powder. The polysaccharide is composed of the molecules of amylose and amylopectin. Each has a varied percentage depending on the source and kind of starch, although amylose and amylopectin generally account for 20–25% and 75–80% of total starch, respectively [33]. Starch, which is present in fruits, seeds, and roots in the form of grains in leaves, tubers, stem core, and rhizomes, is the most significant polysaccharide for storing energy in plants [34,35,36]. Similar to potatoes, rice, wheat, maize, and cassava, it constitutes the majority of the human diet’s carbohydrate intake [37].

### 2.5. Glycogen

Another type of carbohydrate that stores energy is called glycogen. It is similar to other storage polysaccharides like amylopectin [16] but is more compacted and branched. In general, the cytoplasm of animal cells contains glycogen, which is a type of glucose. Although glycogen is essential for physiological metabolism, it has no industrial use and is only included here for completeness [38].

### 2.6. Chitin

Chitin is a linear polysaccharide obtained from animal that is extremely hydrophobic and includes acetyl and amino groups inside its unit. It is insoluble in water and conventional organic solvents, but it is soluble in specialized solvents such as dimethylacetamide with 5% lithium chloride and chloroalcohols in aqueous mineral acid solutions. Another frequent structural polysaccharide, it may be found in fungi’s mycelia and spores, as well as the exoskeletons of insects, crustaceans, and other invertebrates [39,40].

### 2.7. Hyaluronic Acid

Hyaluronic acid is one type of glycoprotein, known as mucopolysaccharides or mucins. In comparison to other polysaccharides like heparin, it is one among the few polysaccharides discovered in vertebrate tissue and is more frequently found in developing embryos. These are polysaccharides that have been covalently bonded to proteins. Proteoglycans are one of the others. Hyaluronic acid, frequently known as hyaluronan, is a straight-chain polysaccharide with a molecular mass of about 7 × 10^6^ g/mol that is frequently tightly linked to proteins by a hydrogen bond such those of water [41]. As a result, extraction and isolation are difficult tasks. It has certain biological applications, such as being used in surgical applications with alginate to improve wound healing [42,43].

### 2.8. Alginate

The seaweed alginate is a long-chain hydrophilic polymer that gives flexibility and strength to the cell walls of the algae. Since 600 B.C., it has been used as food.

However, it was not until 1896 that Akrefting was able to effectively extract pure alginate from seaweed. For the first time, alginate was used as a stabilizing component in ice cream by the company Kelco in 1929, and it became a commercially accessible product [40]. Alginate is a safe and natural food ingredient. Alginate has excellent biological properties compared to other seaweed polysaccharides, including ion cross-linking, pH sensitivity, biocompatibility, and biodegradability, which have been extensively used in the food and nutraceutical industries. Alginate is the only polysaccharide with carboxyl groups naturally present in each component residue [44].

## 3. Processing and Characterization of Natural Polymers

The two basic steps in polymer processing are the conversion of polymers into final products with the necessary structure, such as scaffolds, microneedles, and food packaging sheets, as well as the conversion of polymers into powders and pellets for additional processing. In contrast to the former, which may include extrusion and mixing, the latter may make use of processes like injection molding or film casting. This chapter will go through these concepts. It is necessary to characterize polymers in order to determine their properties such as mechanical strength, thermal conductivity, microstructure, and density, as well as their utility, quality assurance, and safety [45].

### 3.1. Blends and Composites

Polymers can be employed more widely as fillers or as matrices in blends and composites. A composite is a system made up of two or more different elements linked together to form a multiphase system with many components while the individual elements maintain their physical and chemical identities. A composite is made up of a filler, which may be a woven fabric, metal, ceramic, or polymer fiber, and a polymer matrix. As a result, polymers can serve as a matrix or filler in composites. Because simpler, mono-component synthetic or natural materials cannot give the needed physical and/or physicochemical qualities, these systems are required [14].

Composites can be made from polymers, both synthetic and natural. Natural and synthetic polymers are blended to develop a new breed of materials with more diverse properties that may be used in a wider range of applications. The production of synthetic polymers, while offering advantages like product consistency and ease of production, has also raised environmental concerns due to their nonbiodegradability and potential toxicity, which has led to an increase in the popularity of natural polymer-based composites in recent years. When two or more polymers are physically combined, either while molten or while dissolving in a suitable solvent, blends are created. Different polymer blends can be used to create a variety of structures, including molecular composites, miscible one-phase and separated phase, compatible and incompatible alloys, interpenetrating and semi-interpenetrating polymer networks, and more.

Polymer mixtures are classified into two categories: compatible blends and incompatible blends. Incompatible mixes are immiscible mixtures with distinctly differentiated phases. The mechanical qualities of these blends are often poor. Compatible blends are those that combine to create a single phase in which the individual components are morphologically indistinguishable. Compared to the component polymers, these polymer blends are able to provide excellent mechanical properties. Incompatible polymer mixtures, contrary to popular opinion, are more common (Table 2) [46].

### 3.2. Processing Techniques

The technology used to manufacture polymer composites is determined by a number of factors, including the nature of the fiber and polymer, the intended purpose, which might be for construction or the fabrication of car components, or biological uses such as scaffolding and wound healing.

#### 3.2.1. Extrusion Molding

Extrusion is the most typical method for molding extruded polymers. To obtain homogeneity of the polymer with or without the addition of other polymers and additives, polymers are melted under heat and shear to create blends or composites. To achieve homogeneity and the required shape, polymers must often be processed in their melt form when being used as matrix materials for engineering or medical applications. Injection molding also calls for the polymer to flow into a cavity and cool there, whereas extrusion molding primarily relies on the flow of molten polymer through a screw [51,52].

#### 3.2.2. Solvent Casting

This method is frequently used to create polymer films. Numerous uses, including packaging, transdermal drug delivery, and wound healing, depend on the ability of polymers to form films. Coatings can also be produced using films. One of the most important properties of a film for any application is its consistency. Certain techniques are employed to achieve consistency in the production of films from neat polymers or mixtures. Self-absorption of monolayers (SAM), spin coating, thermal spraying, solvent casting, floating technique, and Langmuir–Blodgett film formation are all techniques for making films. The two most used techniques are solvent casting and spin coating. In this paper, we look into nanocellulose as a polymer composite reinforcement, focusing on the manufacturing processes needed to make such a composite. The polymer matrix is composed of fish skin gelatin, and the reinforcing component is cotton linter cellulose whiskers [53].

#### 3.2.3. Cellulose Nanoparticles

The reduction in particle size to the micro- or nanoscale can significantly enhance the polymer’s functioning properly. Nanocellulose is presently the subject of intensive research with the goal of developing high-performance composites. Some bacteria, such as *Acetobacter* species, create nanocellulose using the bottom-up technique. They can also be produced from plants via a top-down technique that includes the breakdown of plant components [54,55]. Algae and tunicate are other sources of nanocellulose [56,57,58]. 

### 3.3. Characterization

Structural characterization, which determines the conformation of polymers like polysaccharides, is based on an understanding of the usual energy release for particular types of linkages, as determined by the angle of rotation about the linkage. This works especially effectively for compounds with well-known conformations, such as poly- and oligosaccharides. A polysaccharide or oligosaccharide chain’s exact geometry can be determined, for instance, by understanding the dihedral angles of rotation of the monosaccharide links [59].

A polysaccharide having different rotations at each monosaccharide link, for example, is anticipated to have a random structure. The possibility of a random conformation is constrained by interactions between and within chains because they providing less potential for independent rotation. Conformation is essential for the polymer to work effectively. Consider cellulose and amylose as two polysaccharides with identical monomer units and that are (14)-d-linked poly-d-glucans. However, the helix of amylose is swaying, but the zigzag chain conformation of cellulose is stretched. Because cellulose and amylose have distinct conformations, this is inedible and water insoluble. Polymer characterization also aids in identifying a polymer’s crystalline or amorphous nature. Compounds that crystallize often have much stronger structures. Unlike proteins, which can maintain their crystal structures even in solutions, polysaccharides rarely form crystal structures [59]. X-ray diffraction, magnetic resonance, Fourier transform infrared spectrometry, and small-angle X-ray scattering are some of the techniques used in this section.

#### 3.3.1. Small-Angle X-ray Scattering (SAXS)

This approach is based on X-rays and has an angle that is small. Proteins [60] and polysaccharides [33] are examples of polymers that SAXS can analyze rapidly in solution. The principles of reciprocal law, which link the distance r in a real space with the scattering vector q on a scattering space also referred to as the Fourier space, form the basis of this procedure [33].

SAXS is a rapid low-resolution structural characterization technique for polymers that can also be used with different methods. The other parameters may be determined using the several mathematical models for the SAXS profile that are available, provided that the properties of the X-ray being used are known [60]. SAXS has the advantage of being able to investigate macromolecules in solution and not just crystallized samples. This is especially advantageous for naturally occurring polymers that are difficult to crystallize.

#### 3.3.2. Nuclear Magnetic Resonance (NMR)

Since NMR spectroscopy is site-specific, an analysis can focus on side chains, main chains, and functional groups at specific sites. It is possible to learn about the molecular motion and the polymer’s time-dependent structure. NMR provides superior sensitivity for microscopic structure in a short-range order as compared to other methods like X-ray scattering; nevertheless, information is not well maintained and may be lost on the long-range and higher order. It is difficult for NMR to establish the spatial position of atomic groups. In comparison to more rapid methods like SAXS, NMR also requires a significant amount of running time [33].

#### 3.3.3. X-ray Diffraction

X-ray diffraction can be used to determine the phases, occurrence of amorphous structure, degree of crystallinity, and crystalline microstructure of a polymeric material. The interference and diffraction of X-ray beams while they leave a crystal constitute the basis of the X-ray diffraction principle. Moreover, XRD may provide additional details about a material, like the orientation of a polymer or the filler inside it. For instance, the characteristics of polypropylene with a biaxial orientation differ significantly from those with other orientations. While a polymer sample is crystallizing, it is also possible to measure it in instantaneously. This could theoretically make it feasible to regulate processing parameters. To obtain high-quality readings from XRD, the sample needs to be well-oriented and crystalline. Samples for XRD analysis can be prepared using a number of methods. For instance, starch films and powders are prepared by drying and conditioning using a desiccator before XRD assessment [61].

## 4. Cellulose and Starch Extraction, Purification, and Modification

The term “polymer” means “many pieces” (it is derived from the Greek words poly, which means “many,” and meros, which means “parts”). Large molecules known as polymers have molar weights ranging from dozens to millions. Synthetic polymers like plastics, material filaments, and specialty rubbers make up around 80% of the organic chemicals market. By combining numerous small molecules into one large molecule, polymers are created. Small molecules known as monomers are utilized to create polymers. Expanding polymers are made from monomer units that are specifically joined together, whereas building polymers are made from monomer units that join together in such a way that a small molecule, generally water, is provided during each reaction. Polymers are abundantly dispersed in nature. There are several natural polymers, including proteins, cellulose, gelatin, starch, chitin, and chitosan, are found in the bodies of plants, animals, and humans. These polymers can be isolated, refined, and modified to improve their performance in a wide range of applications [62].

As alternatives to petroleum-based polymers, cellulose and starch show potential in this field. These materials are widely distributed around the world and, because they are extracted from natural sources, are renewable [63]. Starch is inexpensive, widely available, and biodegradable [64]. Because of its accessible accessibility, low density, biodegradability, sustainability, and strong thermo-mechanical qualities, cellulose is a material that shows great promise. Furthermore, because cellulose is derived from natural sources, it is innocuous [65].

### 4.1. Extraction, Purification, and Modification of Cellulose

Natural cellulose-based bioproducts have difficulties dispensing with synthetic plastics, even though the bioplastics business is expanding quickly due to numerous innovative discoveries. These challenges include difficulties with scalability, a high production cost, and—above all—the limited function of cellulosic materials. However, cellulosic materials need to meet performance standards and have qualities suitable for the end user in order to compete synthetic plastics. In this regard, surface modification of prefabricated cellulosic materials improves the range of applications for cellulose while preserving its chemical profile, mechanical properties, and biodegradability. Many chemical modifications have been made to cellulose to maximize its potential especially for specific fields of interest [66].

#### 4.1.1. Cellulose Extraction

The cellulose -glucopyranosyl residues are linked together by 1 → 4 links. Cellulose crystallizes into monoclinic, rod-shaped crystals. The unit cell’s long b-axis is constructed by chains that are oriented parallel to the fiber direction. It is assumed that the chains are pleated to facilitate the creation of intrachain hydrogen bridges between O-4 and O-6 and between O-3 and O-5. While hydrophobic interactions are present along the c-axis, intermolecular hydrogen bridges along the a-axis stabilize the parallel chains. A total of 60% of the original cellulose can be found in the crystalline sections. Amorphous gel patches that can crystallize when moisture is removed divide these sections. These areas appear to include the linkages that are acid- or alkali-labile as well. These bonds are hydrolyzed, resulting in the formation of microcrystalline cellulose. The molecular weight of this partially depolymerized cellulose product is 30–50 kD, although it lacks a fibrose structure. It is nonetheless water insoluble [67]. 

#### 4.1.2. Cellulose Modification

Cellulose constitutes the majority of the cell walls in all plants. It has a crystalline structure and is a complex carbohydrate. In contrast to starch, which contains primarily −1,4 linkages, cellulose possesses *ß*-1,4-glycosidic bonds that all of the glucose is comprised. Cotton and wood fibers are the two main sources of raw materials for the manufacture of cellulosic plastics. Alkali and carbon disulfide are used to dissolve plant fibers, resulting in viscose that is then transformed into cellulose in the form of cellophane in a sulfuric acid and salt sulfate solution [68].

Due to their high strength and stiffness, biodegradability, and renewability, as well as their production and use in composites, cellulose macro- and nanofibers are becoming increasingly prevalent. A relatively recent field of research is the use of cellulose nanofibers in the production of composites. Cellulose macro- and nanofibers are suitable for usage as reinforcement in composite materials due to their superior mechanical, thermal, and biodegradation properties. Because cellulose fibers are naturally hydrophilic, increasing their surface roughness is critical for producing composites with enhanced properties [69].

### 4.2. Starch Extraction, Purification, and Modification

With applications in a wide range of industries, including adhesive and binding, paper production, corrugating, construction, coatings and paints, chemical, pharmaceuticals, textiles, oilfield, food, and feed, starch is a versatile and extensively used ingredient [70]. The temperature at which gelatinization occurs, the quantity of water absorbed in relation to temperature, pasting capacity, gel strength, and other properties are specific to each starch source. Since there are very few accessible sources of starches, the creation of modified starches is a widely used alternative that can enhance the qualities of native starches and aid them to escape some of their limitations, thus expanding their applications in industry [71].

#### 4.2.1. Extraction Methods of Starch

Several plant organs use starch as a common form of stored carbohydrate. Being part of numerous meals makes it the most significant source of carbohydrates in the human diet. Starch and its by-products are also important for industry, particularly in the paper and textile industries. The sources are typically concentrated in areas where starch is isolated. Wheat, potatoes, maize and potatoes produce 99% of the world’s starch, in both natural and modified forms. Certain alternative starches are also accessible commercially. Starches produced from legumes (peas, lentils) have recently gained interest due to properties that appear to make them a viable alternative for chemically modified starches in a number of commodities [67].

Various foods and commercial goods include rice starch as an additive. Desserts and bakery goods are among of the preferred uses of processed meals owing to the inherent benefits of rice starch, which include its small and uniform size distribution, white color, and clean odor. However, rice protein binds strongly to the starch granules’ surface in the endosperm, which raises the cost of starch separation compared to other starches. Rice protein can be removed from rice flour using alkaline solvents, surfactants, or protein hydrolyzing enzymes [72]. Protein extraction for starch separation frequently makes use of alkaline solvents like NaOH and surfactants like sodium lauryl sulfate (SLS) and dodecylbenzene sulfonate (DoBS). These solvents induce the oligomeric protein structures to separate and change into soluble forms. For isolating rice starch, an aqueous 1.2% DoBS solution with 0.12% sodium sulfite outperformed 0.2% NaOH or 1.2% SLS including 0.12% sodium sulfite. The effectiveness of protein removal may be increased even more by repeating rapid extraction processes (1–2 h) with new solution. The protein extractability ascended lightly but the starch loss increased significantly; hence, raising the extraction temperature was not advised. The paste’s ability to adhere to surfaces had a substantial impact on the remaining protein content of rice starch; less protein made the paste viscous and reduced the pasting temperature [73].

#### 4.2.2. Modification of Starch

Through physical and chemical processes, the characteristics of starch, amylose, and amylopectin can be improved or “tailored” to suit or adapt to a particular application or food product. The amorphous component rises when starch granules are broken down by grinding or applying pressure at various water contents, boosting dispersibility and swellability in cold water, lowering the temperature at which gelatinization occurs by 5 to 10 °C, and increasing enzymatic vulnerability. For instance, amylase breakdown and water absorption are both greater and higher in bread dough formed from flour with damaged starch. Extruded starches have a lower viscosity, are more soluble, and dispersible. Chemical changes occur at temperatures ranging from 185 to 200 °C, as evidenced by the partial breakdown of amylase when heated correctly. Other sugars found in addition to maltose were isomaltose, gentiobiose, sophorose, and 1,6-anhydroglucopyranose [67].

## 5. Biological Applications of Natural Polymers

### 5.1. Anti-Inflammatory Activity

Natural polysaccharides are frequently utilized in nanotechnology for the treatment of inflammatory diseases [74], and their anti-inflammation properties have been investigated [75]. For example, TCM polysaccharides mainly block chemotactic and adhesion factor expression as well as the activity of key enzymes involved in the inflammatory process [76]. While other polysaccharides inhibit inflammatory-related mediators like cytokines (IL-1b, IL-6, TNF-), NO (nitric oxide), and NO (nitric oxide), and reduce inflammatory cell infiltration, sulfated polysaccharides derived from algae have an anti-inflammatory effect by preventing leukocyte migration to the sites of inflammation [77]. Due to their potential therapeutic benefits, nutritional supplements derived from plants and animals have been intensively explored in the development of new drugs [78]. Natural products, their derivatives, and biomacromolecules constitute around 50 percent of the current drugs on the market [79]. These supplements are widely recommended because of their variety of chemical components and lack of side effects compared to synthetic varieties. 

Certain plants include natural substances that may have the ability to act as NSAID-like anti-inflammatory agents and be used as osteoarthritis drugs. Curcumin, capsaicin, berberine, sinomenine, rapamycin, white willow bark, and other natural substances all exhibit proven pharmacological effects [80].

### 5.2. Hypoglycemic and Hypocholesterolemic Activities

It is possible to treat hyperglycemia, hyperlipidemia, hyperinsulinemia, and insulin resistance with ganoderma atrum polysaccharide, and it can also assist in avoiding kidney damage in type 2 diabetics. Since the 1980s, polysaccharides have been extensively researched for their hypoglycemic and hypocholesterolemic effects in clinical trials. To increase the therapeutic efficacy of loaded proteins and increase their stability, natural polysaccharides can be employed as nanocarriers. Orally administered insulin-loaded dextran–chitosan nanoparticulate polyelectrolyte complex had a better bioavailability and a more prolonged hypoglycemic impact [12]. Additional examples of polysaccharides having hypoglycemic and hypocholesterolemic properties include chitosan, kefiran, and sulfated polysaccharides from Bullacta exarate [81,82,83].

According to a number of studies, *α*-glucosidase and *α*-amylase are crucial for the insulin regulation. As a result, it is believed that inhibiting these enzymes is a good choice for treating diabetes. Inhibiting these enzymes can, in fact, limit carbohydrate digestion of and delay the absorption of glucose [84]. In this context, acarbose and plant extracts (which may include numerous biologically active substances) have been described as hypoglycemic drugs that exhibit α-glucosidase inhibitory action and can reduce the blood glucose level (BGL) [85].

Concerning actions that decrease cholesterol, it is generally recognized that dietary intake and cholesterol production, absorption, and secretion frequently have an impact on plasma cholesterol levels, especially LDL-c. In fact, several studies have reported evidence that different dietary proteins and their hydrolysates can improve blood lipid profiles; for instance, diets with soy [86], milk proteins [87], and fish proteins [88].

### 5.3. Anticoagulant Activity

Sulfated polysaccharides called unfractionated and low molecular weight heparins are employed as anticoagulants; however, they have side effects include bleeding and thrombocytopenia [89]. Among the several polysaccharide properties, anticoagulant action has received much attention. Polysaccharides, particularly sulfated polysaccharides, have been shown to exhibit biological effects such as anti-tumor, antioxidant, and anticoagulant characteristics [81]. The anticoagulant action of these sulfated polysaccharides is significantly influenced by the high sulfate concentration [90]. Natural polysaccharides from a variety of marine sources, including corals, marine fungi, microalgae, and shellfish (shrimp, crab, squilla, lobster, and crayfish) [91], may be regarded as anticoagulant agents [92].

### 5.4. Antiviral Activity

The ability of sulfated polysaccharides from seaweeds to block the replication of enveloped viruses such as the herpes simplex virus (HSV), human immunodeficiency virus (HIV), human cytomegalovirus, dengue virus, and respiratory syncytial virus has been known since the 1950s [77]. Sulfated exopolysaccharides, which have a crucial biological role as antiviral agents, can be produced by a variety of microalgae species [90]. Chinese traditional medicine-derived polysaccharides have been utilized as antiviral medications for a long time because they can boost and strengthen the immune system by stimulating macrophagocytes to enhance their phagocytic activity and trigger the release of IFN- and antibodies [76]. Biochemical characteristics of microalgal polysaccharides include antioxidant, antibacterial, and antiviral action [93].

## 6. Application of Natural Polymers in Food

The fact that polymers can be branched and that individual monomers might have distinct characteristics, like charge (in the case of polyelectrolytes) and hydrophobicity (in the case of amphipathic polymers), adds to the complexity. Proteins and polysaccharides are the main types of food polymers. Several polysaccharides, including starch, cellulose, chitosan, galactomannans, carrageenans, alginates, agars, inulins, pectins, xanthans, and gums, are abundant in nature and important sources of nutrition. They are also frequently employed as necessary bulk food. Furthermore, polysaccharides might be utilized as significant food additives because of their remarkable and occasionally exceptional properties which include thickening, stabilizing, gelling, and emulsifying agents. Polysaccharides (i.e., starches, celluloses), polyamides (proteins), and minor amounts of polynucleotides (DNA, RNA) are the three principal types of naturally occurring polymers that make up the majority of meals [94].

Proteins are linear complex heteropolyelectrolytes that have a distinctive sequence of up to 20 different amino acid monomers with a variety of charge, hydrophobicity, and aromatic structural configurations. In live cells and animals, protein, which has an average degree of polymerization of about 103, performs a variety of biological functions. Meals typically contain some form of protein, which also serves as a source of amino acids for our own metabolism. They have the ability to produce and stabilize emulsions, foams, and gels, as well as enhance viscosity and water holding capacity. Despite their use, proteins are more expensive than other natural biopolymers, particularly starches and celluloses, which restricts their use in technology. Some of the key components include gelatin, milk proteins, egg proteins, and plant proteins [95].

Heteropolymers of sugars and their derivatives are known as polysaccharides. Many are polyelectrolytes that can be linear or branched. Polysaccharides generally have a degree of polymerization between 103 to 104. ”Nutritional” functions, according to biological processes, serve as energy storage for metabolism (in particular starch in plants and glycogen in animals) and “building material” (such as cellulose in plants and chitin in fungus cell walls and the exoskeletons of arthropods like crustaceans). The latter category, known as structural polysaccharides, is composed mostly of mixed and very complex structures and occurs in several forms (Table 3) [96]. 

Starch and protein are both essential dietary components that are rich in macronutrients. This section will concentrate on natural polymers as food additives and components in food formulations having a specialized purpose beyond basic nutritional value.

These specific applications of natural polymers in food may be divided into two basic categories:

- Stabilizing food microstructures through gelling, thickening, emulsion, and foaming as well as using processing aids including cryoprotectants to increase freeze–thaw stability, drying aids, and encapsulant material.
- Additional physiological and biological functionality, such as those provided by functional foods with specific health claims including lowering blood cholesterol levels, raising satiety, enhancing bioavailability, and inhibiting microbial growth [97].

### Regulatory Aspects

Natural polymers that are classified as either additives or ingredients, with the bulk falling under the additive category. Gelatin, on the other hand, is categorized as an ingredient. Ingredients are listed by name in the product’s list of ingredients, whereas additives must be identified by a distinctive number (the E-number in the European Union and the INS number abroad) and are optionally identified by name. In the US, additives are governed by the JECFA (Joint/WHO Expert Committee on Food Additives) and the European Commission. The legislation is used to protect consumer health and to ensure ethical behavior in the food sector [98].

The goal of additives regulation is to establish acceptable ingestion limits and purity criteria for diverse compounds. An acceptable daily intake (ADI) and an international number (INS, International Number System) or an E-number are assigned to the additive when it has been approved. The INS/E-number validates its acceptability. The European Food and Safety Agency (EFSA) is in charge of monitoring health claims made for natural polymers used as additives in the EU. The EFSA assesses whether or not the scientific evidence currently available for the health claim is sufficient. In the EU, only EFSA-approved health claims may be advertised on food packaging. Insufficient research (RCTs are preferred), inadequate investigations, or insufficient test subjects used in the studies result in the majority of applications being refused. Many natural polymers, however, passed EFSA evaluation and had their health claims approved (Table 4) [99].

## 7. Pharmaceutical Applications of Natural Polymers

Natural polymers are used for a wide range of applications in both the polymer and pharmaceutical industries. Because the pharmaceutical industry is so extensive, there is a continual need to consider different applications. As a result, knowing how natural polymers are utilized in the pharmaceutical sector would logically benefit the polymer business in recognizing the broader uses of these polymers and adding the essential requirements to fulfil the end applications (e.g., give varied functions). The pharmaceutical industry’s polymer applications are largely focused on medication delivery systems. The following section of this chapter discusses drug entrance sites into the body and gives an outline of the potential applications of natural polymers.

### 7.1. Transdermal Drug Delivery Devices

Polymers are commonly used in transdermal medicine administration procedures. They function as crucial packaging components, coatings, penetration enhancers, ease of handling drug device handling, structural support for the device in the form of a backing layer, and control drug release rate control. They are frequently used in transdermal patches because of their distinctive properties. The development of transdermal drug delivery device components from more readily accessible natural polymers becomes essential as petrochemical-based resources for the manufacturing of synthetic polymers grow more expensive and scarcer. Following a description of the various components of the transdermal drug delivery system, the usage of natural polymers in each element, the matrix, adhesive layer, rate-controlling membrane, backing layer, release liner, and penetration improver will be discussed. Polymers are used more than any other material in transdermal drug delivery (TDD) because they contain features that are significant in the drug delivery process [100]. They can help with pharmaceutical release control from carrier formulations [101]. Silicones, polyvinyl alcohol, chitosan, polyacrylates, and polyesters including PLGA, PELA, and PLA, and cellulose derivatives are among the polymers often used in TDD.

Natural polymers are the ideal choice in TDD because they are easily available, inexpensive, potentially biodegradable, and biocompatible. They may also be treated to various chemical and surface modifications to match the requirements of the TDD system. A TDD system combines one or more polymers with an embedded medication for regulated and sustained drug delivery into or through the skin [102]. High analytical product yields demand for the chemical inertness and purity of polymers utilized in TDD systems. Additionally, it must have physical properties that are sufficient for the intended use. The material must be suitable for processing and must not degrade. In addition, because a TDD patch system would be in touch with a person’s skin for a prolonged period of time, biodegradability and safety are paramount properties to include in the design [103].

### 7.2. Natural Polymers in Transdermal Drug Delivery

Polymers have been used in transdermal medicine delivery since the 1980s. The drug is to be absorbed into the skin from a matrix of cross-linked linear polymer chains seen in the majority of transdermal patches [102]. Transdermal drug delivery polymers include silicones, chitosan, polyvinyl alcohol, polyvinylparrolidone, polyacrylates, and cellulose derivatives. Both natural and synthetic polymers have been used as matrices, gelling agents, emulsifiers, penetration enhancers, and adhesives in transdermal delivery systems [104]; for example, use as in demonstrated successful transdermal testosterone delivery to lab rats using a silicone elastomer synthetic polymer matrix. Another group studied the use of pectin hydrogels for insulin transdermal delivery. Diabetes-prone rats with type 2 diabetes mellitus were administered insulin-loaded pectin hydrogels [105]. Diabetes-prone rats with type 2 diabetes mellitus were administered insulin-loaded pectin hydrogels. The outcomes demonstrated that the transdermal patch effectively and pharmacologically administered insulin across the skin in a dose-dependent manner; [106,107] studied the use of natural polymers such as rubber latex as the adhesive for the backing layer in nicotine patches. As a result, more research into the potential of natural polymers for transdermal medication administration is required.

Natural polymers derived from plants and animals are emerging as a preferable option to synthetic polymers in the development of TDD systems, because they are biocompatible, biodegradable, and degrade into non-toxic monomers, as well as being more easily accessible synthetic polymers are increasingly being employed in the development of TDD systems [2,25]. Synthetic polymers derived from petroleum and synthetically altered polypeptides are known to have limited therapeutic uses due to their toxicity and slow biodegradation rates [100,108,109]. Natural-derived polymeric polysaccharides outperformed traditional carriers like polylactic acid (PLA), poly (lactic-co-glycolic acid) (PLGA), and polyvinyl pyrrolidone (PVP) in terms of drug delivery due to their high-water retention, lack of toxicity, good biocompatibility, biodegradability, and several other important biological properties. For example, sodium alginate could be used for drug encapsulation by cross-linking with metal ions to prepare nanoparticles and thereby improve skin penetration of drugs [110]; chitosan (CS) has significant slow-release properties for drug delivery [111]; hyaluronic acid (HA) can increase skin hydration and involve cell signaling to promote tissue regeneration and wound healing [112], etc.

### 7.3. Natural Polymers in Topical Delivery Systems

Mucoadhesive microspheres, liposomes, solutions, gels, and mucoadhesive hydrogels are commonly used to deliver drugs through the nose. Starch, chitosan, alginate, dextran, hyaluronic acid, and gelatin are examples of natural polymers utilized for nasal medication administration [113]. The interaction of the drug formulation with the mucin surface is known as mucoadhesion. By increasing the contact duration between the drug formulation and nasal mucosa layers in the cavity, the idea behind mucoadhesion is to enable sustained drug administration in nasal membranes [114]. Mucoadhesion reduces the chance of complete mucociliary clearance while promoting drug absorption [115]. Starch is a biodegradable polysaccharide that may easily be converted into microspheres [116]. Examples of starch-loaded intranasal medications in the development stage include insulin [114], morphine [117], inactivated influenza [118], and salbutamol [119]. Chitosan, a naturally occurring polysaccharide with mucoadhesive characteristics, exhibits excellent adhesion to nasal epithelial cells and the overlying mucus layer [120].

Compared to chitosan, PLA, and carboxymethyl cellulose, alginate is a divalent cation-induced rapid gelation, natural polysaccharide with higher mucoadhesion strength [115]. Usually, mucoadhesive polymers are combined with alginate gel to increase the vehicle’s strength and drug-loading efficiency [121].

**Figure 7:**
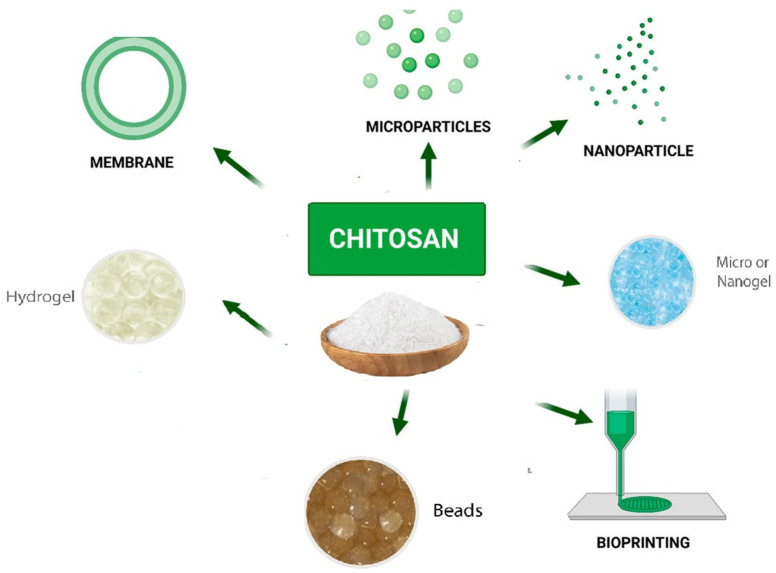
Chitosan-based drug delivery systems [131].

Polysaccharides have recently gained a great deal of interest in the area of tissue engineering [122], cosmetics, and wound healing [123]. They are also extensively applied as drug carriers, building blocks for drug delivery, bioactive materials, and excipients to increase drug delivery [124]. Natural polysaccharides are a potential candidate for numerous uses, including the delivery of medications and vaccinations, due to their versatility in structuring and modification to achieve specific goals. Microorganism-derived polysaccharides including scleroglucan, gellan gum, and xanthan gum have all been intensively researched for utilization in drug delivery [125]. Drugs can be incorporated into bioadhesive polysaccharide nanoparticle carriers to improve their absorption [19]. Pectin, guar gum, amylose, inulin, dextran, chitosan, and chondroitin sulfate are other naturally occurring polysaccharides that have been studied for their potential to be employed as pharmaceutical excipients and for colon-specific drug release [126]. In addition, chitin and chitosan are low-immunogenic and tissue compatible polysaccharides that have demonstrated effectiveness in the delivery of drugs and vaccines [127], as well as the transport of pharmaceuticals to the colon as prodrugs or coating tablets [128]. Chitosan is one of the biopolymers that has been the most widely used as a drug and vaccine delivery system in many preparations (Figure 7) [129]. This is due to the wide variety of antigen that could be encapsulated under mild conditions and without using organic solvents, which avoids the degradation and denaturation of the antigen during processing or after loading [130].

### 7.4. Natural Polymer Implants

Natural polymer uses in implants are particularly appealing for the same reasons that every other biomedical application has been (Table 5) [132]. Because these materials will be embedded in the body for extended periods of time, they must be biocompatible and functional for the entire time that they will be there until they are removed or degraded into the body and release non-toxic, biocompatible degradation products that can be safely eliminated from the body.

## 8. Environmental Impact of Natural Polymers

“Natural” and “green” are not equivalent terms. As their name signifies, natural polymers, often referred to as biopolymers, are substances that are produced by biological organisms such as cellulose, silk, chitin, protein, and DNA. Examples of natural polymers include silk, cellulose, chitin, and protein. It is possible to artificially create natural polymers from natural raw materials in a larger scale. Bio-based polymers are an essential part of the bioeconomy. In 2019, their total production volume reached 3.8 million tons (Mt), representing 1% of the volume of petrochemical polymers produced, with the exclusion of natural rubber plus cellulosic fibers [139,140,141]. The demand for natural polymers in the US is anticipated to increase 6.9% annually [141]. The term “green chemistry,” which first originated in the 1990s, refers to the production of green polymers, also known as sustainable or green chemistry. The International Union of Pure and Applied Chemistry (IUPAC) defines “green chemistry” as “the design of chemical products and processes that decrease or eliminate the use or creation of compounds harmful to humans, animals, plants, and the environment [142]. The concept of bio-based feedstocks is not new in the chemical industry. At least since the start of the twenty-first century, they have proven widely applicable in industries. Bio-based products, however, were not prioritized for a very long time because the price of oil was so low and the growth of oil-based products offered so many chances. The limited availability and variability of fossil fuel materials, environmental concerns, and technological advancements are among many of the causes that have pushed the creation of bio-based polymers and products. Building a chemical industry based on fossil fuels took more than a century. The bio-based polymer sector is quickly catching up with the fossil fuel-based chemical industry, despite its remarkable growth since the late 1990s [142]. 

Because of advancements in white biotechnology, the production of bio-based polymers and other chemicals from renewable resources is now a possibility. First-generation techniques mainly focused on food resources like corn, starch, rice, etc., to produce bio-based polymers. As the discussion over food vs. fuel intensified, technology began to concentrate on cellulose-based feedstocks, including waste from the food, wood, and paper sectors, as well as stems and leaves and solid municipal waste streams. More and more of these technologies are being developed to supplement those previous waste streams; however, it may take a few decades to fully develop the variety of chemicals based on these technologies. Examination of the motivations behind the green and renewable polymer sectors is required in further detail. Rolf Mülhaupt of the University of Freiburg in Germany states that the growth of the green polymer sector is unstoppable because, at the turn of the century, renewable polymers are enjoying a renaissance and there is a significant push towards the development of macromolecular materials derived from biological sources [143].

The proposed transition from petrochemistry to bioeconomy is motivated by a number of factors.

To achieve the desired transition from petrochemistry to the bioeconomy. Economically, the small quantity of oil is expected to bring up the price of oil even further, especially given the expected growth in global energy consumption. For no other reason than that Earth’s population is rapidly expanding, there is an increase in energy consumption on a global level. The affordability and competitiveness of plastics might be significantly impacted. By switching the production of chemical raw materials to other non-renewable resources like coal, the synthesis of plastics could be safeguarded from the projected future oil problem. The need for sustainable and “green” products is therefore made even more important by the growing public concern about global warming. The creation of ecologically friendly products with a reduced carbon footprint is also being accelerated by a wave of environmental laws and regulations [144].

Green manufacturing practices for polymers include the following:

- High percentage of raw materials in the product;
- Clean (waste-free) and efficient production methods;
- The reduction in greenhouse gas emissions;
- Avoiding using additional chemicals, such as organic solvents;
- High manufacturing energy efficiency;
- A product with a high raw material content;

### 8.1. Renewable Polymers

**Figure 8:**
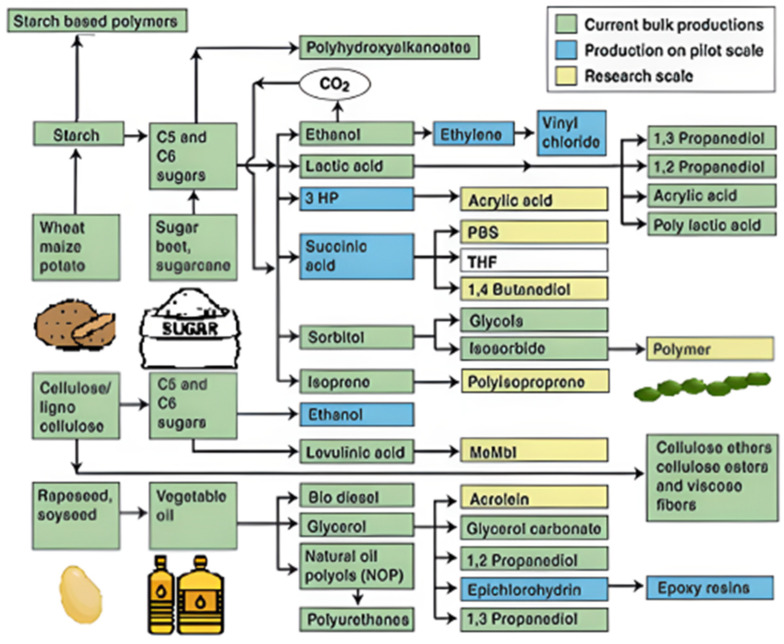
Renewable materials platform [145].

Polymerization of either naturally occurring biopolymers or bio-based monomers can be used to create sustainable polymers. Examples of biomaterials that experienced chemical modification to meet polymer manufacturing and uses include proteins, terpenes, polysaccharides, and polyesters (Figure 8) [145].

Starch and cellulose are the most widely used biopolymers in nonfood industrial applications due to their affordability and accessibility. On the other hand, significant scientific and commercial success has come from research and development efforts into the use of bio-sourced monomers as polymer building blocks or as carbon sources to make biodegradable polymers via a fermentation process. Lactic acid, for example, is a monomer that may be polymerized to poly (lactic acid) via bacteria-fermenting starch [146]. In the same way, a variety from polyesters and polyamides employ succinic acid, which is generated through fermentation, as a monomer as copolymer [147]. Another use of bioresourced polymer is the copolymerization or polymerization of fatty acids produced from lipids for various polymer applications [148,149].

To create derivatives with desired qualities for different applications, the hydroxyl functional group in cellulose may be partly or completely esterified or nitrated [150,151]. Acid hydrolysis has gained a lot of attention in recent times for its potential for extracting the crystalline components of cellulose for use in materials, creating more affordable, effective methods of producing them as a result. A semi-crystalline polymer, starch is composed of approximately 1000–2,000,000 glucose monomers connected by α-1,4 glycosidic linkages [152]. The glucose unit in the starch chain contains three reactive hydroxyl groups: two secondary and one primary hydroxyl group. These groups can serve as anchor points for modifying chemistries.

#### 8.1.1. Polylactic Acid

Despite being discovered in 1845, polylactic acid (PLA) was not commercialized until the beginning of 1990. PLA is a member of the aliphatic polyester family and its primary ingredient is lactic acid. Hydroxyl carboxylic acid, the monomer of lactic acid, is generated by bacteria from corn starch or sugars supplied from renewable resources. Corn has the advantage of offering a high-quality feedstock for fermentation that yields a high-purity lactic acid, which is required for an efficient synthesis process, even though alternative renewable resources may be used. Depending on the microorganism utilized, the fermentation process results in either L-lactic acid or D-lactic acid [153].

PLA is a commercially desirable polymer because it has some similarities to hydrocarbon polymers like polyethylene terephthalate in several ways (PET). High rigidity, exceptional transparency, a glossy appearance, and the capacity to withstand a variety of processing conditions are just some of its distinctive qualities. The ease of melt processing has made it possible to synthesize PLA fibers, which are becoming increasingly used in a variety of textiles, including furniture, curtains, soft nonwoven baby wipes, and tough landscape fabrics. These textiles can perform better than conventional fabrics composed of synthetic substitutes. Implants used for cell growth employ bioresorbable scaffolds made of PLA and different PLA compositions. PLA has been approved by the FDA for a few particular human clinical trials. Additionally, bone support splints have been made using PLA-based polymers [154].

#### 8.1.2. Bio-Polyethylene

Polyethylene (PE) is an essential technological polymer that was previously derived from fossil resources. Ethylene is polymerized under pressure, heat, and the presence of a catalyst to create PE. Either steam cracking of naphtha or other heavy oils or ethanol dehydration traditionally yield ethylene. Currently, microbial PE, also known as green PE, is made from ethanol produced by microbial fermentation. The idea of creating PE from bioethanol is not especially novel. Braskem produced bio-PE and bio-PVC from bioethanol throughout the 1980s. However, the technique was unappealing at the time due to low oil prices and the limits of the biotechnology procedures [155].

**Figure 9:**
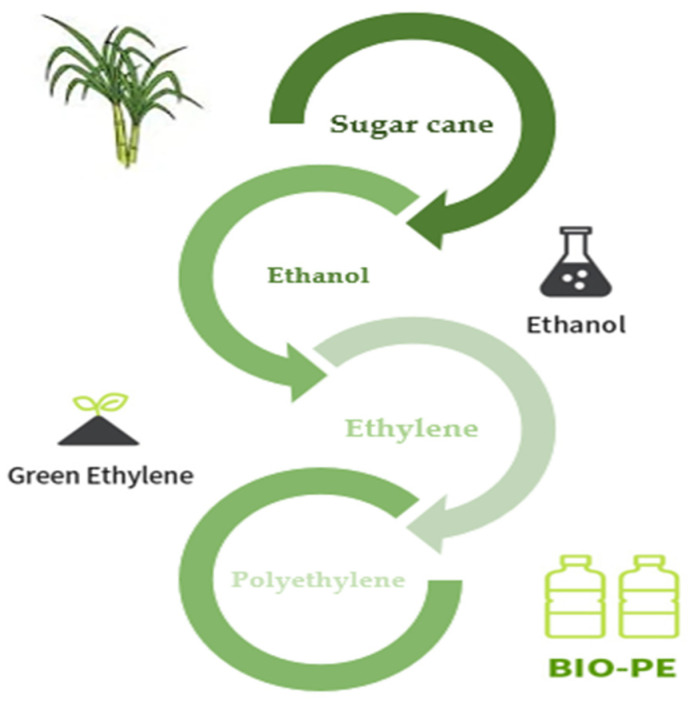
Process for green-PE production [157].

Sugarcane is now used to make bio-PE on an industrial scale from bioethanol. Through a microbial strain and biological fermentation process, bioethanol may also be produced from biorenewable feedstocks such as sugar beet, starch crops like maize, wood, wheat, and other plant wastes. Traditionally, extracted sugarcane juice with a high sucrose concentration is anaerobically fermented to create ethanol. An azeotropic mixture of ethanol + water with a high concentration of the former is produced when ethanol is distilled to remove water. To create ethylene and eventually polyethylene, ethanol is thoroughly dehydrated at high temperatures over a solid catalyst (Figure 9) [156].

#### 8.1.3. Alginates

Alginates are used in several industrial applications as viscosifiers, stabilizers, and agents that form gels, films, or water-binding polymers. Printing on fabrics, ceramics, welding rods, and water treatment are among the applications [158,159,160]. The polymer dissolves in cold water and forms thermostable gels. Custard creams and restructured food are two examples of food products that use these properties. Alginates are also used as stabilizers and thickeners in a variety of drinks, ice creams, emulsions, and sauces [161].

## 9. Economic Impacts of Natural Polymers

The body’s natural extracellular matrix (ECM) is mostly composed of natural polymers such collagen, elastin, and fibrinogen. In the production of a range of medications, natural materials such as protein, enzymes, muscle fibers, polysaccharides, and gummy exudates are used efficiently. Well-known natural polymers utilized in the pharmaceutical industry and other fields include chitosan, carrageenan, ispaghula, acacia, agar, gelatin, shellac, guar gum, and gum karaya. These natural polymers are popular in the pharmaceutical business as emulsifying agents, adjuvants, and packaging adhesives; they are also suitable for the production of pharmaceutical and cosmetic products [162].

Synthetic polymer contamination in the environment has reached catastrophic proportions in developing countries. Petroleum-based plastics are not readily destroyed by microorganisms, and as a result, they build up in the environment. Additionally, there has been a significant rise in oil prices recently. These features have piqued people’s interest in biodegradable polymers. In the 1980s, the first biodegradable plastics and polymers were introduced. The requirement for polymers made from renewable resources has increased significantly over the past 20 years, largely due to two important factors: first, environmental concerns, and, second, the realization that our petroleum supplies are limiting. Synthetic and natural polymers are just two of the elements that can be used to create biodegradable plastics. Natural polymers can be obtained in significant numbers from renewable sources, as compared to synthetic polymers, which are made from non-renewable petroleum resources. Polymer erosion occurs when hydrolytically or enzymatically sensitive linkages in polymeric biomaterials are cleaved. A variety of biodegradable polymers have lately been developed, as have numerous microbes and enzymes capable of breaking them down [163].

According to studies, the United States leads the global market for natural polymers. Africa suffers behind despite global trends toward the production of natural polymers and expanding markets in Latin America, Europe, and Asia. Over the course of several decades, substitutes for cotton and natural rubber have decreased trade profits for developing nations and their comparative advantages in global industry. The development of natural polymers has had an impact on the scientific, technical, and technological fields of agriculture, medicine, pharmaceuticals, and the packaging industry, as well as the economies of countries, primarily in the West, through a variety of cutting-edge materials used in food and beverage, healthcare, and personal care products [164].

### 9.1. Certain Important Natural Polymers for Economy

**Figure 10:**
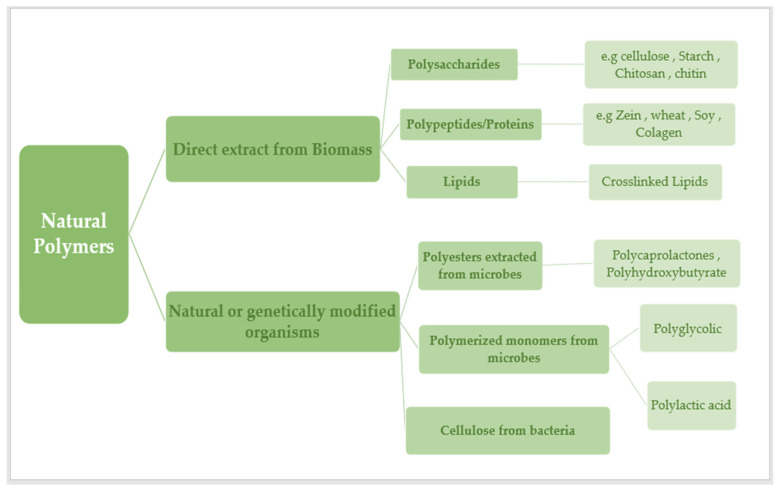
Schematic presentation of bio-based polymers based on their source and production process [167].

Biodegradable polymers are divided into groups, among other criteria, based on their chemical structure, place of origin and synthesis, processing techniques, economic relevance, and usage. Depending on where they are derived from, biodegradable polymers can be divided into two groups: natural polymers (obtained from natural resources) and synthetic polymers (made from oil). Figure 10 depicts the global production capacity of various biopolymers in 2016 [165].

It should be noted that China, the United States, and Sweden held the dominant positions in the global market for natural polymers, accounting for nearly 56% of the market. When the exports of Italy, France, Germany, and Netherlands were included, the combined share of those three countries in the global market exceeded 80%. The analysis of the natural polymer export dynamics in 2018 and 2019 reveals that a number of developing countries, as well as those transitioning to a market economy (countries in transition), demonstrated exceptionally strong export growth. These countries include Mauritius, Azerbaijan, the Russian Federation, Ecuador, Cyprus, Namibia, and Georgia. This might be explained by the way their exports have developed dynamically as a result of numerous multinational firms choosing to locate their production facilities in these nations. With over half (50%) of the global market share for natural polymers in 2019, the EU (27), stands among the top players in this sector of the economy. Three countries, namely Sweden, France, and Italy, led the EU market for natural polymer exports, accounting for 31.3% of all EU exports. Next in line for top exporters of natural polymers in the EU were these eight nations: Germany, Netherlands, Spain, Denmark, Czech Republic, Belgium, and Poland (tied with Ireland) [166].

### 9.2. Industry Use of Natural Biodegradable Polymers

Biopolymers, also known as natural polymers, are polymers that come naturally during all stages of organism development. Activated monomers, which are often created inside of cells through intricate metabolic processes, are most frequently used in enzyme-catalyzed chain growth polymerization reactions in their creation (Table 6) [164].

Numerous products, including plastic bags, automobile parts, and medicinal applications, can be made using biopolymers. Another advantage of using biodegradable materials is that, once they have served their intended use, they can be composted alongside organic waste or used to create biomass. Furthermore, recyclable materials may be recycled by microbes to create valuable monomers and oligomers, which can then be used to create the original products [168].

The automotive industry is one area where the usage of biopolymer materials has an advantageous economic effect. Traditional glass fibers, which are frequently employed as reinforcements, are abrasive and quickly degrade processing equipment. Because flax fibers have a less coarse texture, processing equipment persists for more time [169]. The advantage of natural fibers over synthetic ones, according to Williams and Pool [170], is that the former is more widely available and less costly. An encouraging trend for Canada’s agriculture sector is the growing use of the fiber of flax in auto components, especially for diversification purposes.

Transport pallets have been reinforced by the use of China reed fiber, which is a different sort of natural fiber reinforcement. Making the choice to use China reed pallets was a wise financial move because they require fewer resources and less time to recover their costs than traditional pallets while being just as mechanically stable. China reed pallets are more cost-effective logistically than traditional pallets since they require less fuel to transport due to their lighter mass [171].

The biopolymer sector is still being developed, but shortly it will be fully competitive with the traditional plastics industry in terms of cost. While biopolymers are now manufactured mostly on a small scale, synthetic plastics are synthesized on a large scale. Researchers and business representatives are being encouraged to devote time to further develop these processes by the low cost of the feedstocks derived from renewable resources [172].

**Table 6:** List of common natural polymers [173].

Natural Polymer	Examples
Polysaccharides	Starch, cellulose, chitin
Proteins	Collagen/gelatin, casein, albumin, fibrinogen,
Polyesters	Poly (hydroxyalkanoates)
Other polymers	Lignin, lipids, shellac, natural rubber

## 10. Future Perspectives

Because of their particular physicochemical, biologic, and degrading characteristics, biodegradable polymers are also attractive materials for biomedical applications. They contribute to the reduction in the emissions of greenhouse gases and the depletion of fossil fuels [174]. Nowadays, biopolymers are produced and used in small quantities all over the world. The food packaging and the bioplastic industries are the principal uses for them. In order to better understand how different types of polymers interact with each other and affect the chemical and physical properties of biomaterials, further research must be conducted to enhance the polymeric properties of these biodegradable substances in the future. The development of drug carrier agents and biopolymer-based bio-implants will have a significant positive impact on the medical sector. Further developments could lead to a revolution in the financial stability of medical implants [175].

### 10.1. Natural Polymer Bases in Gums for Food Applications

Polyisoprenes has been incorporated in food, especially chewing gum. Initially, chewing gums were made from naturally occurring polymer-based materials like gutta and other gums like chicle that may be found in nature. More chewing gum companies started focused on using synthetic polymers to make chewing gums, such as polyethylene, polyvinyl acetate, styrene–butadiene copolymers, and isobutylene-isoprene copolymers, as chewing gum demand became more sophisticated and interest in synthetic polymers increased. Previously, natural guayule rubber was coupled with polyvinyl acetate and hydrogenated vegetable oil as plasticizers, emulsifiers, solvents, and inorganic fillers to make a chewing gum based on natural polymers [6]. However, synthetic polymers are currently used in the great majority of chewing gum products on the market. Chewing gums produced from synthetic polymers are hydrophobic, which causes them to stick to surfaces despite their chosen consistency and capacity to attain desirable qualities. Natural polymers offer more hydrophilic possibilities, enabling the production of non-stick chewing gum from natural substrates like natural rubber. The health risks associated with chewing gum swallowing, whether swallowed on purpose or accidentally, are also expected to be lower with natural polymer-based chewing gums because they are more likely to be ingested. One of the disadvantages of using natural-based polymers for food applications, such as polyisoprene, is the presence of trans and cis isomeric forms and protein residues that may induce allergic reactions. In order to better eliminate these allergens from natural polymers, processing techniques have been improved. As a result, more food products will be utilized [176].

### 10.2. Natural Polymer Trends and Prospects in the Cosmetics Industry

Biopolymers, also known as natural polymers, are polymers that occur spontaneously during all stages of organism development. They are generally created by enzyme-catalyzed chain growth polymerization reactions of activated monomers, which are typically created within cells through complicated metabolic processes [164].

There are numerous examples, but because of economic competitiveness, scientific study into physicochemical or sensory features of such polymer connections has been restricted. Instead, scientists have looked into the possible connections between the rheological characteristics of xanthan gum XG and hydroxypropyl guar (HPG) and their filament-stretching abilities in both pure and mixed aqueous solutions as well as in cosmetic emulsions. Different rheological characteristics between XG and HPG were seen in pure solution. For concentrations below 1% w/w, XG solutions had higher stretchability values than HPG, while at higher concentrations, the filament stretching capabilities of both polymers were comparable. A significant synergistic impact for XG/HPG solution at a 25/75 ratio was seen in mixed solutions regardless of total concentration tested (below 0.5% *w*/*w*) [177].

The effect of the synergy on the stretching characteristics is noteworthy because the interaction between XG and HPG increases the maximum filament length. Polysaccharides also have the benefit of resembling biological macromolecules, such as the extracellular matrix (ECM), which increases their potential for use in cell therapeutic approaches [178].

In recent years, there has been a surge in interest in employing polysaccharide materials in tissue engineering [179]. Future tumor treatments may benefit from the use of polysaccharides derived from mushrooms, such as chizophyllan, lentinan, grifolan, polysaccharide-peptide complex (PSP), and polysaccharide-protein complex (PSK), which can stimulate the immune system and have an anticancer effect [180]. Although they have been used for a very long time, mushrooms have recently become more popular in the treatment of cancer. Polysaccharides’ use will most certainly increase in the future due to their safety. Several additional organic synthesizes and chemical modifications have caused significant pollution, which might be decreased by switching to natural polysaccharides [181]. Polysaccharides have been employed more and more in a variety of ways to regulate the distribution of various drugs, particularly due to their biocompatibility and biodegradability properties [182]. The various functional groups present on polysaccharide structures, including the hydroxyl, amino, and carboxylic acid groups, could be further modified to function as a particular biological tool in a variety of fields, such as vaccine adjuvants, drug carriers, tissue engineering scaffolds, and many other pharmacological activities [166]. Natural polysaccharides like glycogen, cellulose, and starch were engineered onto biologically superior molecules to support their biopharmaceutical candidacy using a variety of techniques, including chemical modification, co-polymer grafting, and atom transfer radical polymerization (ATRP) [183].

Drugs with hydrophobic groups can be conjugated with polysaccharides that have hydrophilic groups, creating amphiphilic prodrugs that can self-assemble into nanostructures with improved water solubility. There are significant limitations and challenges for polysaccharides in the pharmaceutical and biological industries, nevertheless, because they are typically taken from natural sources. Examples include changes in viscosity from batch to batch, microbial contamination, viscosity reduction during storage, thickening, and an uncontrolled rate of hydration. Fortunately, these limitations may be avoided through modifications such grafting, cross-linking, and mixing with other natural, synthetic, and semi-synthetic polymers [184].

In addition to polysaccharides naturally forming in combination with other molecules such as proteins and lipids, polysaccharide diversity in nature adds an additional significant obstacle to the extraction and purification of these polysaccharides. Their separation requires the employment of effective and accurate procedures to avoid co-extraction and contamination with other substances. Furthermore, discovering and fully appreciating the relationship between polysaccharide structure and activity may open up new avenues for utilization in biological and pharmaceutical applications [185].

## 11. Conclusions

The majority of natural biopolymers are polysaccharides, which have a variety of chemical and physical properties that make them a strong candidate for use in numerous biological applications. In comparison to other synthetic polymers, polysaccharides have a number of benefits, such as being secure, affordable, stable, hydrophilic, biocompatible, and biodegradable. Additionally, they can be chemically modified and tailored for particular uses in a variety of applications, such as the creation of pharmaceutical materials, drug release agents, and plasma substitutes. Numerous biological functions of polysaccharides exist, such as immunoregulatory, anti-tumor, anti-virus, anti-inflammatory, antioxidative, and hypoglycemic effects. Polysaccharides have received a lot of attention in recent years, emerging as one of the most successful alternatives to traditional medicine. Carbohydrate-based pharmaceuticals have been proven to be successful in a number of disciplines, but they are not attracting the same level of attention as those based on proteins or nucleic acids. We completed our work by urging more research because there are many unanswered issues and undiscovered biological features of various polysaccharides. Further investigation and characterization of the structural activity relation of polysaccharides is necessary to fully explore their potential applications and gain a better understanding of the precise mechanisms behind polysaccharides’ biological activities.

## Figures and Tables

**Table 1:** List of some polysaccharides from various sources.

Source	Polymer
Cells walls of plants	Pectin
Seeds and roots	Galactomannans
Seaweeds	Carragenans, alginates, agar
Animal cell walls	Hyaluronan
Shells of aquatic animals	Chitin
Wood	Cellulose, lignin, hemicellulose
Skins and bones of animals and scales of fish	Gelatin
Bacteria	Xanthan, hyaluronan, gellan
Fungi	Cardlan, scleroglucan, schizophylla

**Table 2:** Natural/synthetic composites and compatibilizers.

Synthetic Polymer	Natural Polymer	Compatibilizer	Processing Technique	Reference
Polypropylene	Sawdust	Maleic anhydride	Extrusion	[14]
Polypropylene	Wood fibers	Ethylene–propylene or ethylene–propylidene copolymer Maleate polypropylene Calcium stearate	Injection forming	[46]
Low density polyethylene	Lignocellulosic fibers Sawdust	Ionomer polyethylene Maleate polypropylene Low molecular weight polypropylnene Maleic anhydride	Extrusion Injection	[46]
Polyurethane	Mechanical pulp	Isocyanates	Pressing	[46]
Phenol formaldehyde	Lignocellulose	Chemical modified fibers	Pressing	[46]
Polyester + PE + PP	Wood fibers	Phenol resins	Pressing	[46]
Carboxylated Nitrile Rubber	Natural rubber	Maleic anhydride grafted polyisoprene epoxy resin	Roll milling	[47]
Chlorinated Polyethylene	Natural rubber	Maleic anhydrided grafted ethylene propylene diene rubber EPDM-g-MA	Thermal mixing followed by roll milling	[48]
Carboxylated nitrile rubber	Natural rubber	Bis(disopropyl) thiophosphoryl polysulfides	Thermal mixing followed by roll milling	[49]
Poly(lactic acid)	Natural rubber	Poly(lactic acid)- natural rubber tri block copolymer		[50]

**Table 3:** Sources of commercially important hydrocolloids used in the food industry [96].

**Botanical**	Plants	Starch, pectin, cellulose
	Trees	Cellulose
	Tree gum exudates	Gum arabic (acasia), gum tragacanth, karaya
	Seeds	Guar gum, tara gum, locust bean gum,
	Tubers	Konjac mannan (glucomannan), potato starch
**Algal**	Red seaweed	Agar, carrageenan
	Brown seaweed	Alginate
**Microbial**		Xanthan gum, dextran, gellan gum, cellulose
**Animal**		Gelatin, caseinate, whey protein, chitosan

**Table 4:** EFSA-approved health claims for products made with natural polymers.

Claim	Hydrocolloid
Maintenance of normal blood cholesterol concentrations	Beta-glucan, konjacmannan glucomannan, pectins, guar gum
Maintenance or achievement of a normal body weight	Konjacmannan glucomannan
Reduction in postprandial glycemic responses	Beta-glucan, pectins

**Table 5:** Examples of natural polymers used in implants.

Polymer	API/Implant	References
Chitosan	Flurbiprofen	[133]
	Timolol maleaic	[133]
	Thymol	[134]
	Quercetin	[134]
	Dexamethason	[133]
	Vancomycin	[135]
Hyaluronic acid/chitosan multilayer coating	Chitosan Imidazole/siRNA nanoplex	[136]
Chitosan/carbonnanotube	Titanium implant	[137]
Collagen/cellulose	Juca extract	[138]
Agarose	Thymolol	[134]
	Quercetin	
Poly (Lactic acid)	Ibuprofen	[138]
	Acetylsalicylic acid	

## References

[1] Balart R., Garcia-Garcia D., Fombuena V., Quiles-Carrillo L., Arrieta M.P. (2021). Biopolymers from Natural Resources. Polymers.

[2] Sharma K., Singh V., Arora A. (2011). Natural Biodegradable Polymers as Matrices in Transdermal Drug Delivery. Int. J. Drug Dev. Res..

[3] Coviello T., Matricardi P., Marianecci C., Alhaique F. (2007). Polysaccharide Hydrogels for Modified Release Formulations. J. Control. Release.

[4] Ahmad Z., Pandey R., Sharma S., Khuller G.K. (2006). Alginate Nanoparticles as Antituberculosis Drug Carriers: Formulation Development, Pharmacokinetics and Therapeutic Potential. Indian J. Chest Dis. Allied Sci..

[5] Qi L., Xu Z. (2006). In Vivo Antitumor Activity of Chitosan Nanoparticles. Bioorg. Med. Chem. Lett..

[6] Olatunji O., Olubowale M., Okereke C. (2018). Microneedle-Assisted Transdermal Delivery of Acetylsalicylic Acid (Aspirin) from Biopolymer Films Extracted from Fish Scales. Polym. Bull..

[7] Biodegradable Natural Polymers. https://link.springer.com/chapter/10.1007/978-3-319-12478-0_2.

[8] Zhou C.Y., Wang Y., Pan D.D., Sun Y.Y., Cao J.X. (2018). The Effect of ATP Marination on the Depolymerization of Actin Filament in Goose Muscles during Postmortem Conditioning. Poult. Sci..

[9] Rabetafika H.N., Paquot M., Dubois P. (2006). Les Polymères Issus Du Végétal: Matériaux à Proprieties Spécifiques Pour Des Applications Ciblées En Industrie Plastique. Biotechnol. Agron. Soc. Environ..

[10] Pillai O., Dhanikula A.B., Panchagnula R. (2001). Drug Delivery: An Odyssey of 100 Years. Curr. Opin. Chem. Biol..

[11] Zhang F., Xu S., Wang Z. (2011). Pre-Treatment Optimization and Properties of Gelatin from Freshwater Fish Scales. Food Bioprod. Process..

[12] Liu Z., Jiao Y., Wang Y., Zhou C., Zhang Z. (2008). Polysaccharides-Based Nanoparticles as Drug Delivery Systems. Adv. Drug Deliv. Rev..

[13] D’Ayala G.G., Malinconico M., Laurienzo P. (2008). Marine Derived Polysaccharides for Biomedical Applications: Chemical Modification Approaches. Molecules.

[14] Mohammed A.M.A., Yunus N.Z.M., Hezmi M.A., Rashid A.S.A. (2021). Sequestration of Carbon Dioxide Using Ground Granulated Blast Furnaces Slag and Kaolin Mixtures. Glob. Nest J..

[15] Dumitriu S. (2005). Polysaccharides: Structural Diversity and Functional Versatility.

[16] Yui T., Ogawa K. (2004). X-Ray Diffraction Study of Polysaccharides. Polysaccharides.

[17] Pérez S., Mazeau K. (2004). Conformations, Structures, and Morphologies of Celluloses. Polysaccharides.

[18] Allen A.W., Cook J.G., Armbruster M.J. (1984). Habitat Suitability Index Models: Pronghorn.

[19] Kulasinski K., Keten S., Churakov S.V., Derome D., Carmeliet J. (2014). A Comparative Molecular Dynamics Study of Crystalline, Paracrystalline and Amorphous States of Cellulose. Cellulose.

[20] Koch U., Popelier P.L.A. (1995). Characterization of C-H-O Hydrogen Bonds on the Basis of the Charge Density. J. Phys. Chem..

[21] Hebeish A., Guthrie J.T. (1981). The Chemistry and Technology of Cellulosic Copolymers.

[22] Roy D., Semsarilar M., Guthrie J.T., Perrier S. (2009). Cellulose Modification by Polymer Grafting: A Review. Chem. Soc. Rev..

[23] Farrán A., Cai C., Sandoval M., Xu Y., Liu J., Hernáiz M.J., Linhardt R.J. (2015). Green Solvents in Carbohydrate Chemistry: From Raw Materials to Fine Chemicals. Chem. Rev..

[24] Wang M., Sun J., Xu B., Chrusciel M., Gao J., Bazert M., Stelmaszewska J., Xu Y., Zhang H., Pawelczyk L. (2018). Functional Characterization of MicroRNA-27a-3p Expression in Human Polycystic Ovary Syndrome. Endocrinology.

[25] Chang C., Duan B., Cai J., Zhang L. (2010). Superabsorbent Hydrogels Based on Cellulose for Smart Swelling and Controllable Delivery. Eur. Polym. J..

[26] Zielke C., Stradner A., Nilsson L. (2018). Characterization of Cereal β-Glucan Extracts: Conformation and Structural Aspects. Food Hydrocoll..

[27] Hu X., Zhao J., Zhao Q., Zheng J. (2015). Structure and Characteristic of β-Glucan in Cereal: A Review. J. Food Process. Preserv..

[28] Kuki H., Yokoyama R., Kuroha T., Nishitani K. (2020). Xyloglucan Is Not Essential for the Formation and Integrity of the Cellulose Network in the Primary Cell Wall Regenerated from Arabidopsis Protoplasts. Plants.

[29] Li Z., Pan X. (2018). Strategies to Modify Physicochemical Properties of Hemicelluloses from Biorefinery and Paper Industry for Packaging Material. Rev. Environ. Sci. Biotechnol..

[30] Rao J., Lv Z., Chen G., Peng F. (2023). Hemicellulose: Structure, Chemical Modification, and Application. Prog. Polym. Sci..

[31] Chandel V., Biswas D., Roy S., Vaidya D., Verma A., Gupta A. (2022). Current Advancements in Pectin: Extraction, Properties and Multifunctional Applications. Foods.

[32] Basak S., Annapure U.S. (2022). Trends in “Green” and Novel Methods of Pectin Modification—A Review. Carbohydr. Polym..

[33] Kajiwara K., Miyamoto T. (2010). ChemInform Abstract: Progress in Structural Characterization of Functional Polysaccharides. ChemInform.

[34] Zhang J., Zhai A. (2020). Microstructure, Thermodynamics and Rheological Properties of Different Types of Red Adzuki Bean Starch. Qual. Assur. Saf. Crops Foods.

[35] Farooq U., Di Mattia C., Faieta M., Flamminii F., Pittia P. (2021). Colloidal Properties and Stability of Olive Oil-in Water Emulsions Stabilized by Starch Particles. Ital. J. Food Sci..

[36] Bojarczuk A., Skąpska S., Mousavi Khaneghah A., Marszałek K. (2022). Health Benefits of Resistant Starch: A Review of the Literature. J. Funct. Foods.

[37] Tan L., Kong L. (2020). Starch-Guest Inclusion Complexes: Formation, Structure, and Enzymatic Digestion. Crit. Rev. Food Sci. Nutr..

[38] Zhu Y., Delbianco M., Seeberger P.H. (2021). Automated Assembly of Starch and Glycogen Polysaccharides. J. Am. Chem. Soc..

[39] Jolly G., Kuscu M.C., Kokate P., Younis M. A Low-Energy Key Management Protocol for Wireless Sensor Networks. Proceedings of the Proceedings—IEEE Symposium on Computers and Communications.

[40] Kulkarni V., Butte K., Rathod S. (2012). Natural Polymers—A Comprehensive Review. Int. J. Res. Pharm. Biomed. Sci..

[41] Guizzardi S., Uggeri J., Belletti S., Cattarini G. (2013). Hyaluronate Increases Polynucleotides Effect on Human Cultured Fibroblasts. J. Cosmet. Dermatol. Sci. Appl..

[42] Taravel F., Mazeau K., Tvaros¡ka I. (2004). Computer Modeling of Polysaccharide–Polysaccharide Interactions. Polysaccharides.

[43] Oerther S., Le Gall H., Payan E., Lapicque F., Presle N., Hubert P., Dexheimer J., Netter P., Lapicque F. (1999). Hyaluronate-Alginate Gel as a Novel Biomaterial: Mechanical Properties and Formation Mechanism. Biotechnol. Bioeng..

[44] Bi D., Yang X., Yao L., Hu Z., Li H., Xu X., Lu J. (2022). Potential Food and Nutraceutical Applications of Alginate: A Review. Mar. Drugs.

[45] Nafchi A.M., Moradpour M., Saeidi M., Alias A.K. (2013). Thermoplastic Starches: Properties, Challenges, and Prospects. Starch-Staerke.

[46] Cazacu I., Miremont G., Geniaux H., Benard-Laribiere A. (2013). Causality and Preventability of Adverse Drug Reactions of Analgesics. Drug Safety.

[47] Onyeagoro G.N. (2013). Reactive Compatibilization of Natural Rubber (NR)/Carboxylated Nitrile Rubber (XNBR) Blends by Maleic Anhydride-Grafted-Polyisoprene (MAPI) and Epoxy Resin Dual Compatibilizers. Int. Ref. J. Eng. Sci..

[48] Sirisinha C., Saeoui P., Guaysomboon J. (2004). Oil and Thermal Aging Resistance in Compatibilized and Thermally Stabilized Chlorinated Polyethylene/Natural Rubber Blends. Polymer.

[49] Naskar N., Debnath S.C., Basu D.K. (2001). Novel Method for Preparation of Carboxylated Nitrile Rubber-Natural Rubber Blends Using Bis(Diisopropyl)Thiophosphoryl Polysulfides. J. Appl. Polym. Sci..

[50] Chumeka W., Pasetto P., Pilard J.F., Tanrattanakul V. (2014). Bio-Based Triblock Copolymers from Natural Rubber and Poly(Lactic Acid): Synthesis and Application in Polymer Blending. Polymer.

[51] Hummel D.O. (1981). Polymeren–Lehrbuch: Polymer Chemistry–an Introduction. Von RB Seymour und CE Carraher, jr. Marcel Dekker Inc. New York–Basel 1981. XVI, 576 S., Zeichn., geb., SFr. 80,–. Nachrichten Chem..

[52] Sowunmi S., Ebewele R.O., Peters O., Conner A.H. (2000). Differential Scanning Calorimetry of Hydrolysed Mangrove Tannin. Polym. Int..

[53] Santos T.M., Men de Sá Filho M.S., Caceres C.A., Rosa M.F., Morais J.P.S., Pinto A.M.B., Azeredo H.M.C. (2014). Fish Gelatin Films as Affected by Cellulose Whiskers and Sonication. Food Hydrocoll..

[54] Turbak A.F., Snyder F.W., Sandberg K.R. (1983). Microfibrillated Cellulose, a New Cellulose Product: Properties, Uses, and Commercial Potential. J. Appl. Polym. Sci. Appl. Polym. Symp..

[55] Herrick F.W., Casebier R.L., Hamilton J.K., Sandberg K.R. (1983). Microfibrillated Cellulose: Morphology and Accessibility. J. Appl. Polym. Sci. Appl. Polym. Symp..

[56] Preston R.D., Nicolai E., Reed R., Millard A. (1948). An Electron Microscope Study of Cellulose in the Wall of Valonia Ventricosa. Nature.

[57] Belton P.S., Tanner S.F., Cartier N., Chanzy H. (1989). High-Resolution Solid-State 13C Nuclear Magnetic Resonance Spectroscopy of Tunicin, an Animal Cellulose. Macromolecules.

[58] Lee K.Y., Aitomäki Y., Berglund L.A., Oksman K., Bismarck A. (2014). On the Use of Nanocellulose as Reinforcement in Polymer Matrix Composites. Compos. Sci. Technol..

[59] Núñez O., Ikegami T., Kajiwara W., Miyamoto K., Horie K., Tanaka N. (2007). Preparation of High Efficiency and Highly Retentive Monolithic Silica Capillary Columns for Reversed-Phase Chromatography by Chemical Modification by Polymerization of Octadecyl Methacrylate. J. Chromatogr. A.

[60] Putnam D.K., Lowe E.W., Meiler J. (2013). Reconstruction of SAXS Profiles from Protein Structures. Comput. Struct. Biotechnol. J..

[61] Detduangchan N., Sridach W., Wittaya T. (2014). Enhancement of the Properties of Biodegradable Rice Starch Films by Using Chemical Crosslinking Agents. Int. Food Res. J..

[62] David A., Lewandrowski K.U., Josten C., Ekkernkamp A., Clasbrummel B., Muhr G. (1996). Surgical Correction of Talipes Equinovarus Following Foot and Leg Compartment Syndrome. Foot Ankle Int..

[63] Ma L., Hu X., Song L., Chen X., Ouyang M., Billot L., Li Q., Malavera A., Li X., Muñoz-Venturelli P. (2023). The Third Intensive Care Bundle with Blood Pressure Reduction in Acute Cerebral Haemorrhage Trial (INTERACT3): An International, Stepped Wedge Cluster Randomised Controlled Trial. Lancet.

[64] Thakur R., Pristijono P., Scarlett C.J., Bowyer M., Singh S.P., Vuong Q.V. (2019). Starch-Based Films: Major Factors Affecting Their Properties. Int. J. Biol. Macromol..

[65] Muhd Julkapli N., Bagheri S. (2017). Nanocellulose as a Green and Sustainable Emerging Material in Energy Applications: A Review. Polym. Adv. Technol..

[66] Gopakumar D.A., Thomas S., Owolabi F.A.T., Thomas S., Nzihou A., Rizal S., Abdul Khalil H.P.S. (2020). Nanocellulose Based Aerogels for Varying Engineering Applications. Encycl. Renew. Sustain. Mater..

[67] Belitz H.D., Grosch W., Schieberle P. (2009). Food Chemistry.

[68] Brostow W., Datashvili T., Miller H. (2010). Wood and wood derived materials. J. Mater. Educ..

[69] Kalia S., Dufresne A., Cherian B.M., Kaith B.S., Avérous L., Njuguna J., Nassiopoulos E. (2011). Cellulose-Based Bio- and Nanocomposites: A Review. Int. J. Polym. Sci..

[70] Maniglia B.C., Castanha N., Le-Bail P., Le-Bail A., Augusto P.E.D. (2021). Starch Modification through Environmentally Friendly Alternatives: A Review. Crit. Rev. Food Sci. Nutr..

[71] Vanier N.L., El Halal S.L.M., Dias A.R.G., da Rosa Zavareze E. (2017). Molecular Structure, Functionality and Applications of Oxidized Starches: A Review. Food Chem..

[72] Maniñgat C.C., Juliano B.O. (1979). Properties of Lintnerized Starch Granules from Rices Differing in Amylose Content and Gelatinization Temperature. Starch-Stärke.

[73] Lim S.T., Lee J.H., Shin D.H., Lim H.S. (1999). Comparison of Protein Extraction Solutions for Rice Starch Isolation and Effects of Residual Protein Content on Starch Pasting Properties. Starch-Stärke.

[74] Muhamad I.I., Lazim N.A.M., Selvakumaran S. (2019). Natural Polysaccharide-Based Composites for Drug Delivery and Biomedical Applications. Natural Polysaccharides in Drug Delivery and Biomedical Applications.

[75] Mzoughi Z., Abdelhamid A., Rihouey C., Le Cerf D., Bouraoui A., Majdoub H. (2018). Optimized Extraction of Pectin-like Polysaccharide from Suaeda Fruticosa Leaves: Characterization, Antioxidant, Anti-Inflammatory and Analgesic Activities. Carbohydr. Polym..

[76] Chen Y., Yao F., Ming K., Wang D., Hu Y., Liu J. (2016). Polysaccharides from Traditional Chinese Medicines: Extraction, Purification, Modification, and Biological Activity. Molecules.

[77] Jiao G., Yu G., Zhang J., Ewart H.S. (2011). Chemical Structures and Bioactivities of Sulfated Polysaccharides from Marine Algae. Mar. Drugs.

[78] Li G., Lou H.X. (2018). Strategies to Diversify Natural Products for Drug Discovery. Med. Res. Rev..

[79] Newman D.J., Cragg G.M. (2016). Natural Products as Sources of New Drugs from 1981 to 2014. J. Nat. Prod..

[80] Bost J., Maroon A., Maroon J. (2010). Natural Anti-Inflammatory Agents for Pain Relief. Surg. Neurol. Int..

[81] Yu Y., Shen M., Song Q., Xie J. (2018). Biological Activities and Pharmaceutical Applications of Polysaccharide from Natural Resources: A Review. Carbohydr. Polym..

[82] Nayak A.K., Ahmed S.A., Tabish M., Hasnain M.S. (2019). Natural Polysaccharides in Tissue Engineering Applications. Natural Polysaccharides in Drug Delivery and Biomedical Applications.

[83] Moradi Z., Kalanpour N. (2019). Kefiran, a Branched Polysaccharide: Preparation, Properties and Applications: A Review. Carbohydr. Polym..

[84] Bhandari M.R., Jong-Anurakkun N., Hong G., Kawabata J. (2008). α-Glucosidase and α-Amylase Inhibitory Activities of Nepalese Medicinal Herb Pakhanbhed (Bergenia Ciliata, Haw.). Food Chem..

[85] Preetha P.P., Devi V.G., Rajamohan T. (2012). Hypoglycemic and Antioxidant Potential of Coconut Water in Experimental Diabetes. Food Funct..

[86] Lovati M.R., Manzoni C., Gianazza E., Arnoldi A., Kurowska E., Carroll K.K., Sirtori C.R. (2000). Soy Protein Peptides Regulate Cholesterol Homeostasis in Hep G2 Cells. J. Nutr..

[87] O’connor L.E., Berry J.W., Weiss J., Gilbert P. (2002). Guilt, Fear, Submission, and Empathy in Depression. J. Affect. Disord..

[88] Berge R.K., Tronstad K.J., Berge K., Rost T.H., Wergedahl H., Gudbrandsen O.A., Skorve J. (2005). The Metabolic Syndrome and the Hepatic Fatty Acid Drainage Hypothesis. Biochimie.

[89] Costa L.S., Fidelis G.P., Cordeiro S.L., Oliveira R.M., Sabry D.A., Câmara R.B.G., Nobre L.T.D.B., Costa M.S.S.P., Almeida-Lima J., Farias E.H.C. (2010). Biological Activities of Sulfated Polysaccharides from Tropical Seaweeds. Biomed. Pharmacother..

[90] de Jesus Raposo M.F., de Morais A.M.M.B., de Morais R.M.S.C. (2014). Bioactivity and Applications of Polysaccharides from Marine Microalgae. Polysaccharides.

[91] Mohan K., Ravichandran S., Muralisankar T., Uthayakumar V., Chandirasekar R., Seedevi P., Rajan D.K. (2019). Potential Uses of Fungal Polysaccharides as Immunostimulants in Fish and Shrimp Aquaculture: A Review. Aquaculture.

[92] Olennikov D.N., Kashchenko N.I., Chirikova N.K., Koryakina L.P., Vladimirov L.N. (2015). Bitter Gentian Teas: Nutritional and Phytochemical Profiles, Polysaccharide Characterisation and Bioactivity. Molecules.

[93] Peng X., Usman B., Kaushik N., Hoffman J., Wang D., Saenko K. (2017). VisDA: The Visual Domain Adaptation Challenge. arXiv.

[94] Walstra P. (2003). Physical Chemistry of Foods.

[95] Telis V.R.N. (2012). Biopolymer Engineering in Food Processing.

[96] Phillips G.O. (2009). Handbook of Hydrocolloids.

[97] Sayas-Barberá E., Pérez-Álvarez J.A., Navarro-Rodríguez de Vera C., Fernández-López M., Viuda-Martos M., Fernández-López J. (2022). Sustainability and Gender Perspective in Food Innovation: Foods and Food Processing Coproducts as Source of Macro- and Micro-Nutrients for Woman-Fortified Foods. Foods.

[98] Kyriakopoulou K., Keppler J.K., van der Goot A.J. (2021). Functionality of Ingredients and Additives in Plant-Based Meat Analogues. Foods.

[99] Viebke C., Al-Assaf S., Phillips G.O. (2014). Food Hydrocolloids and Health Claims. Bioact. Carbohydr. Diet. Fibre.

[100] Kim J.K., Kim H.J., Chung J.Y., Lee J.H., Young S.B., Kim Y.H. (2014). Natural and Synthetic Biomaterials for Controlled Drug Delivery. Arch. Pharm. Res..

[101] Cleary G.W. (1993). Transdermal Delivery Systems: A Medical Rationale. Topical Drug Bioavailability, Bioequivalence, and Penetration.

[102] Fujimoto T., Enomoto K., Matuo K., Tojo K. (2006). In Vivo Evaluation of Skin Permeability of Drugs after Applying Adhesive Transdermal Patches.

[103] Sirbubalo M., Tucak A., Muhamedagic K., Hindija L., Rahić O., Hadžiabdić J., Cekic A., Begic-Hajdarevic D., Husic M.C., Dervišević A. (2021). 3d Printing—A “Touch-Button” Approach to Manufacture Microneedles for Transdermal Drug Delivery. Pharmaceutics.

[104] Sun Y., Tojo K., Chien Y.W. (1986). Kinetics and Thermodynamics of Drug Permeation through Silicone Elastomers (II) Effect of Penetrant Lipophilicity. Drug Dev. Ind. Pharm..

[105] Musabayane C.T., Munjeri O., Bwititi P., Osim E.E. (2000). Orally Administered, Insulin-Loaded Amidated Pectin Hydrogel Beads Sustain Plasma Concentrations of Insulin in Streptozotocin-Diabetic Rats. J. Endocrinol..

[106] Suksaeree J., Boonme P., Taweepreda W., Ritthidej G.C., Pichayakorn W. (2012). Characterization, in Vitro Release and Permeation Studies of Nicotine Transdermal Patches Prepared from Deproteinized Natural Rubber Latex Blends. Chem. Eng. Res. Des..

[107] Musabayane C.T., Tufts M.A., Mapanga R.F. (2010). Synergistic Antihyperglycemic Effects between Plant-Derived Oleanolic Acid and Insulin in Streptozotocin-Induced Diabetic Rats. Ren. Fail..

[108] Shi W., Dumont M.J., Ly E.B. (2014). Synthesis and Properties of Canola Protein-Based Superabsorbent Hydrogels. Eur. Polym. J..

[109] Deming T.J. (2007). Synthetic Polypeptides for Biomedical Applications. Prog. Polym. Sci..

[110] Jadach B., Świetlik W., Froelich A. (2022). Sodium Alginate as a Pharmaceutical Excipient: Novel Applications of a Well-Known Polymer. J. Pharm. Sci..

[111] Abd El-Hack M.E., El-Saadony M.T., Shafi M.E., Zabermawi N.M., Arif M., Batiha G.E., Khafaga A.F., Abd El-Hakim Y.M., Al-Sagheer A.A. (2020). Antimicrobial and Antioxidant Properties of Chitosan and Its Derivatives and Their Applications: A Review. Int. J. Biol. Macromol..

[112] Zhu J., Tang X., Jia Y., Ho C.T., Huang Q. (2020). Applications and Delivery Mechanisms of Hyaluronic Acid Used for Topical/Transdermal Delivery—A Review. Int. J. Pharm..

[113] Chaturvedi M., Kumar M., Pathak K. (2011). A Review on Mucoadhesive Polymer Used in Nasal Drug Delivery System. J. Adv. Pharm. Technol. Res..

[114] Duan X., Mao S. (2010). New Strategies to Improve the Intranasal Absorption of Insulin. Drug Discov. Today.

[115] Patil S.B., Sawant K.K. (2009). Development, Optimization and in Vitro Evaluation of Alginate Mucoadhesive Microspheres of Carvedilol for Nasal Delivery. J. Microencapsul..

[116] Grinberg O., Gedanken A. (2010). The Development and Characterization of Starch Microspheres Prepared by a Sonochemical Method for the Potential Drug Delivery of Insulin. Macromol. Chem. Phys..

[117] Illum L., Watts P., Fisher A.N., Hinchcliffe M., Norbury H., Jabbal-Gill I., Nankervis R., Davis S.S. (2002). Intranasal Delivery of Morphine. J. Pharmacol. Exp. Ther..

[118] Coucke D., Schotsaert M., Libert C., Pringels E., Vervaet C., Foreman P., Saelens X., Remon J.P. (2009). Spray-Dried Powders of Starch and Crosslinked Poly(Acrylic Acid) as Carriers for Nasal Delivery of Inactivated Influenza Vaccine. Vaccine.

[119] Xu E.Y., Guo J., Xu Y., Li H.Y., Seville P.C. (2014). Influence of Excipients on Spray-Dried Powders for Inhalation. Powder Technol..

[120] Illum L. (2003). Nasal Drug Delivery—Possibilities, Problems and Solutions. J. Control. Release.

[121] Pal D., Nayak A.K. (2012). Novel Tamarind Seed Polysaccharide-Alginate Mucoadhesive Microspheres for Oral Gliclazide Delivery: In Vitro-in Vivo Evaluation. Drug Deliv..

[122] Li Q., Williams C.G., Sun D.D.N., Wang J., Leong K., Elisseeff J.H. (2004). Photocrosslinkable Polysaccharides Based on Chondroitin Sulfate. J. Biomed. Mater. Res. A.

[123] Bragd P.L., Van Bekkum H., Besemer A.C. (2004). TEMPO-Mediated Oxidation of Polysaccharides: Survey of Methods and Applications. Top. Catal..

[124] Ngwuluka N.C. (2018). Responsive Polysaccharides and Polysaccharides-Based Nanoparticles for Drug Delivery. Stimuli Responsive Polymeric Nanocarriers for Drug Delivery Applications: Volume 1: Types and Triggers.

[125] Alvarez-Lorenzo C., Blanco-Fernandez B., Puga A.M., Concheiro A. (2013). Crosslinked Ionic Polysaccharides for Stimuli-Sensitive Drug Delivery. Adv. Drug Deliv. Rev..

[126] Chourasia M.K., Jain S.K. (2004). Polysaccharides for Colon Targeted Drug Delivery. Drug Deliv..

[127] Suginta W., Khunkaewla P., Schulte A. (2013). Electrochemical Biosensor Applications of Polysaccharides Chitin and Chitosan. Chem. Rev..

[128] Sinha A., Sinha M., Burgert S. (2001). Reinfusion of Drained Blood as an Alternative to Homologous Blood Transfusion after Total Knee Replacement. Int. Orthop..

[129] Luo Y., Wang Q. (2014). Recent Development of Chitosan-Based Polyelectrolyte Complexes with Natural Polysaccharides for Drug Delivery. Int. J. Biol. Macromol..

[130] Arca H.Ç., Günbeyaz M., Şenel S. (2009). Chitosan-Based Systems for the Delivery of Vaccine Antigens. Expert. Rev. Vaccines.

[131] Mohammed A.S.A., Naveed M., Jost N. (2021). Polysaccharides; Classification, Chemical Properties, and Future Perspective Applications in Fields of Pharmacology and Biological Medicine (A Review of Current Applications and Upcoming Potentialities). J. Polym. Environ..

[132] Ivanova E.P., Bazaka K., Crawford R.J. (2014). Natural Polymer Biomaterials: Advanced Applications. New Funct. Biomater. Med. Healthc..

[133] Braga M.E.M., Pato M.T.V., Silva H.S.R.C., Ferreira E.I., Gil M.H., Duarte C.M.M., de Sousa H.C. (2008). Supercritical Solvent Impregnation of Ophthalmic Drugs on Chitosan Derivatives. J. Supercrit. Fluids.

[134] Dias A.M.A., Braga M.E.M., Seabra I.J., Ferreira P., Gil M.H., De Sousa H.C. (2011). Development of Natural-Based Wound Dressings Impregnated with Bioactive Compounds and Using Supercritical Carbon Dioxide. Int. J. Pharm..

[135] Yang C.C., Lin C.C., Liao J.W., Yen S.K. (2013). Vancomycin-Chitosan Composite Deposited on Post Porous Hydroxyapatite Coated Ti6Al4V Implant for Drug Controlled Release. Mater. Sci. Eng. C.

[136] Hartmann H., Hossfeld S., Schlosshauer B., Mittnacht U., Pêgo A.P., Dauner M., Doser M., Stoll D., Krastev R. (2013). Hyaluronic Acid/Chitosan Multilayer Coatings on Neuronal Implants for Localized Delivery of SiRNA Nanoplexes. J. Control. Release.

[137] Ahmed R.A., Fekry A.M., Farghali R.A. (2013). A Study of Calcium Carbonate/Multiwalled-Carbon Nanotubes/Chitosan Composite Coatings on Ti-6Al-4V Alloy for Orthopedic Implants. Appl. Surf. Sci..

[138] Ma S.L., Lu Z.W., Wu Y.T., Zhang Z.B. (2010). Partitioning of Drug Model Compounds between Poly(Lactic Acid)s and Supercritical CO_2_ Using Quartz Crystal Microbalance as an in Situ Detector. J. Supercrit. Fluids.

[139] Skoczinski P., Carus M., Tweddle G., Ruiz P., de Guzman D., Ravenstijn J., Käb H., Hark N., Dammer L., Raschka A. (2023). Bio-Based Building Blocks and Polymers: Global Capacities, Production and Trends 2022–2027. Ind. Biotechnol..

[140] Docksai R. (2012). Market for Bioplastics. Futurist.

[141] de Frutos M.Á., Manzano C. (2014). Market Transparency, Market Quality, and Sunshine Trading. J. Financ. Mark..

[142] Mülhaupt R. (2013). Green Polymer Chemistry and Bio-Based Plastics: Dreams and Reality. Macromol. Chem. Phys..

[143] Carus M., Carrez D., Kaeb H., Venus J. (2011). Level Playing Field for Bio-Based Chemistry and Materials.

[144] Gawel E., Pannicke N., Hagemann N. (2019). A Path Transition towards a Bioeconomy—The Crucial Role of Sustainability. Sustainability.

[145] Brostow W., Datashvili T., Jiang P., Miller H. (2016). Recycled HDPE Reinforced with Sol-Gel Silica Modified Wood Sawdust. Eur. Polym. J..

[146] Sun Q., Mekonnen T., Misra M., Mohanty A.K. (2016). Novel Biodegradable Cast Film from Carbon Dioxide Based Copolymer and Poly(Lactic Acid). J. Polym. Environ..

[147] Adkins J., Pugh S., McKenna R., Nielsen D.R. (2012). Engineering Microbial Chemical Factories to Produce Renewable “Biomonomers”. Front. Microbiol..

[148] Rajput S.D., Hundiwale D.G., Mahulikar P.P., Gite V.V. (2014). Fatty Acids Based Transparent Polyurethane Films and Coatings. Prog. Org. Coat..

[149] Maiti B., De P. (2013). RAFT Polymerization of Fatty Acid Containing Monomers: Controlled Synthesis of Polymers from Renewable Resources. RSC Adv..

[150] Klemm D., Heublein B., Fink H.P., Bohn A. (2005). Cellulose: Fascinating Biopolymer and Sustainable Raw Material. Angew. Chem. Int. Ed..

[151] Trinh B.M., Mekonnen T. (2018). Hydrophobic Esterification of Cellulose Nanocrystals for Epoxy Reinforcement. Polymer.

[152] Copeland L., Blazek J., Salman H., Tang M.C. (2009). Form and Functionality of Starch. Food Hydrocoll..

[153] Erwin P., Pohlen M., Beckman J.E. (2008). The Outer Disks of Early-Type Galaxies. I. Surface-Brightness Profiles of Barred Galaxies. Astron. J..

[154] Dorozhkin S.V. (2009). Calcium Orthophosphate-Based Biocomposites and Hybrid Biomaterials. J. Mater. Sci..

[155] Rinderer T.E., Harris J.W., Hunt G.J., De Guzman L.I. (2010). Breeding for Resistance to Varroa Destructor in North America. Apidologie.

[156] Chen G., Li S., Jiao F., Yuan Q. (2007). Catalytic Dehydration of Bioethanol to Ethylene over TiO2/γ-Al2O3 Catalysts in Microchannel Reactors. Catal. Today.

[157] http://www.sojitz.com/en/news/2012/07/20120705.php.

[158] Teli M.D., Chiplunkar V. (1986). Role of Thickeners in Final Performance of Reactive Prints. Text. Dye. Print..

[159] Qin Y., Cai L., Feng D., Shi B., Liu J., Zhang W., Shen Y. (2007). Combined Use of Chitosan and Alginate in the Treatment of Wastewater. J. Appl. Polym. Sci..

[160] Xie Z.P., Huang Y., Chen Y.L., Jia Y. (2001). A New Gel Casting of Ceramics by Reaction of Sodium Alginate and Calcium Iodate at Increased Temperatures. J. Mater. Sci. Lett..

[161] Timothy F., Devinder M., Iain H. (2009). F-35 Joint Strike Fighter Structural Prognosis and Health Management an Overview. ICAF 2009, Bridging the Gap between Theory and Operational Practice: Proceedings of the 25th Symposium of the International Committee on Aeronautical Fatigue, Rotterdam, The Netherlands, 27–29 May 2009.

[162] Sell S.A., Wolfe P.S., Garg K., McCool J.M., Rodriguez I.A., Bowlin G.L. (2010). The Use of Natural Polymers in Tissue Engineering: A Focus on Electrospun Extracellular Matrix Analogues. Polymers.

[163] Holland N.D., Clague D.A., Gordon D.P., Gebruk A., Pawson D.L., Vecchione M. (2005). “Lophenteropneust” Hypothesis Refuted by Collection and Photos of New Deep-Sea Hemichordates. Nature.

[164] Anyika M., Gholami H., Ashtekar K.D., Acho R., Borhan B. (2014). Point-to-Axial Chirality Transfer—A New Probe for “Sensing” the Absolute Configurations of Monoamines. J. Am. Chem. Soc..

[165] Patwary S., Maraz K.M., Shahida S., Ahmed A., Khan R.A. (2021). A Review on the Properties and Applications of Biodegradable Polymers. GSC Adv. Res. Rev..

[166] Dziuba R., Grabowska K., Wawro D., Wietecha J., Wysokińska Z. (2021). Natural Polymers on the Global and European Market-Presentation of Research Results in the Łukasiewicz Research Network—Institute of Biopolymers and Chemical Fibers-Case Studies on the Cellulose and Chitosan Fibers. Autex Res. J..

[167] Kukoyi A.R. (2015). Economic Impacts of Natural Polymers. Nat. Polym..

[168] Samir A., Ashour F.H., Hakim A.A.A., Bassyouni M. (2022). Recent Advances in Biodegradable Polymers for Sustainable Applications. Npj Mater. Degrad..

[169] Stamboulis A., Baillie C.A., Garkhail S.K., Van Melick H.G.H., Peijs T. (2000). Environmental Durability of Flax Fibres and Their Composites Based on Polypropylene Matrix. Appl. Compos. Mater..

[170] Williams G.I., Wool R.P. (2000). Composites from Natural Fibers and Soy Oil Resins. Appl. Compos. Mater..

[171] Corbière-Nicollier T., Gfeller Laban B., Lundquist L., Leterrier Y., Månson J.A.E., Jolliet O. (2001). Life Cycle Assessment of Biofibres Replacing Glass Fibres as Reinforcement in Plastics. Resour. Conserv. Recycl..

[172] Kolybaba M., Tabil L.G., Panigrahi S., Crerar W.J., Powell T., Wang B. (2013). Biodegradable Polymers: Past, Present, and Future. ASABE/CSBE North Central Intersectional Meeting.

[173] Percival S.L., Thomas J., Linton S., Okel T., Corum L., Slone W. (2012). The Antimicrobial Efficacy of Silver on Antibiotic-Resistant Bacteria Isolated from Burn Wounds. Int. Wound J..

[174] Narancic T., Cerrone F., Beagan N., O’Connor K.E. (2020). Recent Advances in Bioplastics: Application and Biodegradation. Polymers.

[175] Tarazona N.A., Machatschek R., Balcucho J., Castro-Mayorga J.L., Saldarriaga J.F., Lendlein A. (2022). Opportunities and Challenges for Integrating the Development of Sustainable Polymer Materials within an International Circular (Bio)Economy Concept. MRS Energy Sustain..

[176] Farber T.M., Clewell A.E., Endres J.R., Hauswirth J., Van Gemert M., Schauss A.G., Sheane C.A. (2010). Safety Assessment of a Novel Ingredient for Removable Chewing Gum. Food Chem. Toxicol..

[177] Jamshidian M., Jalal S., Jansen C. (2014). MissMech: An R Package for Testing Homoscedasticity, Multivariate Normality, and Missing Completely at Random (MCAR). J. Stat. Softw..

[178] Darge H.F., Andrgie A.T., Tsai H.C., Lai J.Y. (2019). Polysaccharide and Polypeptide Based Injectable Thermo-Sensitive Hydrogels for Local Biomedical Applications. Int. J. Biol. Macromol..

[179] Tchobanian A., Van Oosterwyck H., Fardim P. (2019). Polysaccharides for Tissue Engineering: Current Landscape and Future Prospects. Carbohydr. Polym..

[180] Pandya U., Dhuldhaj U., Sahay N.S. (2019). Bioactive Mushroom Polysaccharides as Antitumor: An Overview. Nat. Prod. Res..

[181] Maciel J.V., Durigon A.M.M., Souza M.M., Quadrado R.F.N., Fajardo A.R., Dias D. (2019). Polysaccharides Derived from Natural Sources Applied to the Development of Chemically Modified Electrodes for Environmental Applications: A Review. Trends Environ. Anal. Chem..

[182] Layek B., Mandal S. (2020). Natural Polysaccharides for Controlled Delivery of Oral Therapeutics: A Recent Update. Carbohydr. Polym..

[183] Gopinath V., Saravanan S., Al-Maleki A.R., Ramesh M., Vadivelu J. (2018). A Review of Natural Polysaccharides for Drug Delivery Applications: Special Focus on Cellulose, Starch and Glycogen. Biomed. Pharmacother..

[184] Saidin N.M., Anuar N.K., Meor Mohd Affandi M.M.R. (2018). Roles of Polysaccharides in Transdermal Drug Delivery System and Future Prospects. J. Appl. Pharm. Sci..

[185] Van Dam J.E.G., Van Den Broek L.A.M., Boeriu C.G. (2017). Polysaccharides in Human Health Care. Nat. Prod. Commun..

